# Antisense Oligonucleotides and Small Interfering RNA for the Treatment of Dyslipidemias

**DOI:** 10.3390/jcm11133884

**Published:** 2022-07-04

**Authors:** Clarice Gareri, Alberto Polimeni, Salvatore Giordano, Laura Tammè, Antonio Curcio, Ciro Indolfi

**Affiliations:** 1 Division of Cardiology, Department of Medical and Surgical Sciences, Magna Graecia University, 88100 Catanzaro, Italy; clarice88@hotmail.it (C.G.); polimeni@unicz.it (A.P.); sasigiordano@gmail.com (S.G.); tamme@unicz.it (L.T.); curcio@unicz.it (A.C.); 2 Mediterranea Cardiocentro, 80138 Naples, Italy

**Keywords:** hypercholesterolemia, miRNA, ASO, siRNAF

## Abstract

The burden of atherosclerotic disease worldwide necessitates implementing the treatment of its risk factors. Among them, hypercholesterolemia has a central role. In addition to conventional small organic compounds and the recently introduced monoclonal antibodies, new technologies are arising such as the antisense oligonucleotides and small interfering RNAs (siRNAs) that operate upstream, blocking the mRNA translation of the proteins specifically involved in lipid metabolism. In this review, we briefly explain the mechanisms of action of these molecules and discuss the difficulties related to their in vivo use as therapeutical agents. We go over the oligonucleotides tested in clinical trials that could potentially revolutionize the care of patients by acting on proteins involved in the lipoprotein metabolism and regulation, namely: angiopoietin-like protein 3 (ANGPTL3); lipoprotein a (Lp(a)); apolipoprotein B (Apo B); apolipoprotein C III (Apo C-III); and proprotein convertase subtilisin–kexin type 9 (PCSK9). Finally, the differences between ASOs and siRNAs, their future possible clinical applications, and the role of Inclisiran, a siRNA direct against PCSK9 to reduce LDL-C, were reviewed in detail.

## 1. Introduction

Cardiovascular disease (CVD) includes a broad group of disorders affecting both the heart and the blood vessels. Despite the effort to improve drug treatments for prevention, CVD remains the leading cause of mortality worldwide, taking about 17.9 million lives each year according to WHO records [1]. Therefore, there is a need to find innovative therapies that can slow CVD progression. In this scenario, the prevention and treatment of risk factors such as diabetes, obesity, hypertension, and hypercholesterolemia become fundamental. Hypercholesterolemia in particular has been the focus of extensive research due to its association with an elevated risk of developing atherosclerotic cardiovascular disease (ASCVD). Indeed, the demand for more effective therapies led to the development of new drugs in the past decade, mainly aiming to lower lipid levels in the bloodstream. The conventional approach to treating dyslipidemia is based on small organic compounds such as statins, which inhibit enzyme and receptor function in nanogram to microgram quantities [2], and ezetimibe, a nonstatin drug that reduces intestinal cholesterol absorption. These drugs are taken by oral administration; however, their half-lives are relatively short, with the subsequent need for daily administration. Additionally, although they are effective at lowering cholesterol, they may lead to adverse effects such as muscle pain, elevated CK enzymes, and, rarely, rhabdomyolysis/myositis. To overcome the limitations of statins, different pharmacological approaches have been developed in addition to dietary restrictions. After describing the effects of mutations in the PCSK9 gene on LDL-C and ASCVD, therapeutic approaches including monoclonal antibodies (e.g., alirocumab, evolocumab) and small interfering ribonucleic acid (siRNA) (e.g., inclisiran) were developed to modulate and inhibit PCSK9. Even though these drugs are quite effective, their main limitation is their high cost.

More recently, bempedoic acid, a novel oral nonstatin drug for lowering low-density lipoprotein cholesterol (LDL-C), is a prodrug that reduces cholesterol synthesis by inhibiting adenosine triphosphate citrate lyase, an enzyme upstream from 3-hydroxy-3-methylglutaryl-coenzyme. Recent studies showed it to be effective and safe in combination with statins for lowering LDL-C, and it has the potential to reduce the risk of muscle-related adverse events, which can limit the utilization and effectiveness of statin therapy [3]. Nevertheless, a discrete residual risk for CVD remains even with the newer classes of medicines [4,5,6,7]. For these reasons, the search for new optimal therapies to reduce lipid concentration is still ongoing. An innovative approach is to operate upstream, blocking the mRNA translation of proteins specifically involved in lipid metabolism; this would allow for targeting grams of protein with a single molecule of inhibitor, allowing for extended dosing intervals (up to 6 months). Two main classes of molecules have been used lately for this aim: antisense oligonucleotides (ASO) and double-stranded short interfering RNA (siRNA) [8]. Perhaps promising new classes of non-coding RNA (i.e., long ncRNA, miRNA) known for playing a role in CVD risk could be used; hence, studying these classes of molecules and their mechanisms opens the possibility of gaining leverage over CVD development.

Table 1 presents a comprehensive table describing the different characteristics of the drugs employed in the treatment of dyslipidemias.

### 1.1. Antisense Oligonucleotides (ASO)

**Figure 1:**
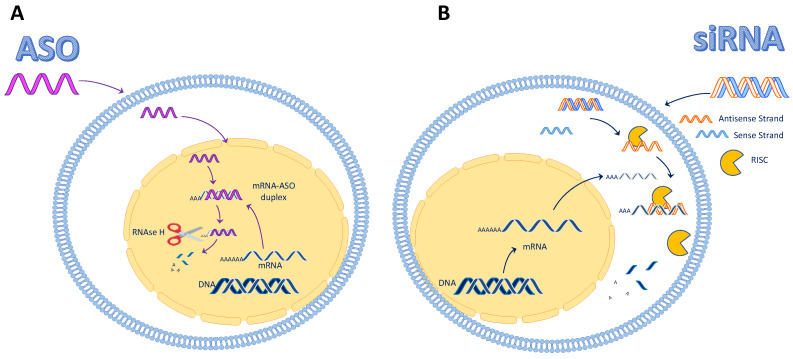
mRNA degradation mechanisms through ASO and siRNA. (**A**) The ASO mechanism of action: The single-strand ASO enters the cell and the nucleus; once it is bound to the mRNA, the double-strand siRNA is recognized by RNAse H and degraded. (**B**) The siRNA mechanism of action: The double-strand siRNA enters the cell; in the cytoplasm, the duplex opens and the antisense strand binds to the RNA-induced silencing complex (RISC). The mRNA is recognized by the antisense-RISC complex and degraded.

ASOs have been studied since the 1980s in different fields; in 1978, a report showed the efficacy of using a short synthetic oligonucleotide to inhibit Rous sarcoma virus 35S RNA translation [9]. Twenty years later, in 1998, Ion Pharmaceutical saw its first compound, fomivirsen (Vitravene), approved by the US FDA. Since then, we have witnessed hundreds of ASO-based drugs tested in clinical trials, with at least 18 companies (including multinationals like Sanofi, Pfizer, and Bristol-Myers Squibb) sponsoring the research on developing more efficient ASOs [10]. First-generation ASOs were long (usually 12–25 bases) synthetic single-strand nucleotides that took advantage of an endogenous mechanism to prevent protein translation. In particular, ASOs recognize in a Watson and Crick base pairing a complementary sequence on the mRNA target, leading to RNAse–H activation and subsequent mRNA degradation [11] (Figure 1). More recently, newer ASOs have also been designed to block translation (i.e., peptide nucleic acids (PNAs), O-methyl antisense oligonucleotides (OMOs)) and modulate the splicing of oligomers that do not recruit RNAse H.

**Figure 2:**
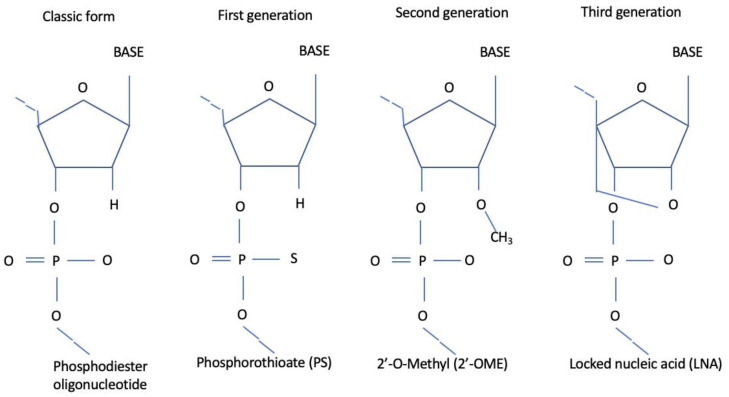
Different ASO generations.

The first-generation ASOs have longer half-lives and a greater resistance to nucleases compared with classic phosphodiester oligonucleotides. Optimization was obtained by replacing one of the non-bridging oxygen atoms in the phosphate group with either a sulfur group (phosphorothioates), a methyl group (methylphosphonates), or amines (phosphoramidates) [12]. They are able to activate RNAse H, although they produce undesired aspecific side effects such as immune stimulation and complement activation [12]. To overcome those weaknesses, several improvements have been made, leading to the creation of second- and third-generation ASOs. There are four possible strategies for modifying an ASO: chemically modifying some nucleotides, changing the sugars, acting on the phosphodiester linkage, or altering the backbone [13]. Second-generation ASOs have an alkyl modification at the 2′ position of the ribose, and they are most commonly 2′-O-methyl (2′-OME) and 2′-O-methoxyethyl (2′-MOE) ASOs. They show an increased RNA binding affinity [14], higher resistance to nuclease degradation (i.e., Gapmers) [15], and lower immunostimulatory activity [16] compared with the first generation [12]. Third-generation modifications were made to improve the nuclease stability and target the affinity and pharmacokinetic profiles of the ASOs. Locked nucleic acids (LNAs), peptide nucleic acids (PNAs), morpholino compounds, and hexitol nucleic acids (HNAs) are the most common modifications employed. They act differently by causing the steric hindrance of the ribosomal machinery, resulting in translational arrest [12] (Figure 2).

### 1.2. Short Interfering RNA (siRNA)

siRNAs are sequences of double-stranded RNA with lengths of 21–23 nucleotides that use a miRNA-mediated silencing mechanism; miRNA are short endogenous sequences, typically 18–25 nucleotides, that bind the 3′UTR of the mRNA target, driving the RNA-induced silencing complex (RISC)-mediated RNA silencing. Upon cellular incorporation, the sense strand is removed. The antisense binds to the RISC, driving the complex to its target; once the target mRNA is recognized, the complex starts the cleavage, creating mRNA fragments that are fully degraded by other cellular nucleases [17,18,19]. siRNAs can also operate differently by identifying only partially complementary mRNA sequences; this binding leads to the sequestration of mRNA in the P-bodies, with the subsequent inhibition of the translation [18] (Figure 1). Even though it is a rare event, siRNAs can regulate translation by directly targeting chromatin [20]. Interestingly, siRNAs can also directly interact with their sisters, miRNAs, inhibiting their function and causing an upregulation of the translation of the mRNA targeted by the miRNA [21,22].

Unlike ASOs, which live and operate as a single strand, siRNAs remain bound to the RISC, targeting multiple RNA copies, thereby having a long-term effect [23]. The first study on siRNA-induced gene silencing demonstrated how a siRNA could suppress both endogenous and exogenous genes in different mammalian cell lines [24]. Since then, siRNAs have been studied as an exciting tool for gene silencing and have undergone several chemical modifications to improve their performance. Unlike ASOs, siRNAs are exceptionally stable; however, unmodified siRNAs are not very active [25]. A first modified siRNA has been successfully tested against HBV in mice; these duplex sense and antisense strands lack 2′-OH groups and are differentially modified, leading to increased efficacy and stability [26]. Several other modification patterns have been tested [25], making the second generation of siRNAs particularly useful for progressing towards clinical use [27], primarily those related to reducing siRNAs immunogenicity. Indeed, especially when associated with a lipidic or polycation-based delivery system, siRNAs can induce a potent innate immune response [28]. Introducing 2V-O-methyl (2VOMe) uridine or guanosine nucleosides into the siRNA duplex is well tolerated and still sufficient for inducing strong gene silencing [28,29]. This finding is remarkable because on the one hand, a robust immune activation could be helpful [30,31]; on the other hand, it could limit dose administration [28].

### 1.3. Specific Cell Target Delivery of ASOs or siRNAs In Vivo

Synthetic small oligonucleotides have revolutionized functional genomics because of their reliability, efficacy, and simplicity. However, ASO/siRNA applications in therapeutics have not followed the same pace; a lack of efficient delivery strategies has reduced the initial optimism. Indeed, to be effective, synthetic oligonucleotides must reach their RNA target inside the cytoplasm. Once they have entered the body, oligonucleotides must pass through several barriers: serum nuclease degradation, rapid excretion via the kidney or sequestration by spleen and liver, slow diffusion in extracellular binding, and inefficient endocytosis by specific cells. Even after all these steps, when the oligonucleotides reach the appropriate cell target, they need to be released from the endocytic vesicles still in their active form [32]. Hence, both naked ASOs and siRNAs are limited by their pharmacokinetics [33], and the cellular uptake and release into the cytoplasm of synthetic oligonucleotides have been a bottleneck in the clinical application of these molecules for years. Encouragingly, thanks to the new advances in technologies, synthetic nucleotides’ performance can be adjusted by careful engineering. Delivery technologies are mainly based on encapsulation in nanoparticles or conjugation with ligands, even if viral vectors have also been tested [34].

ASOs are usually administered in saline and rely on their structure for uptake. They can bind several cell-surface receptors [35,36]. Furthermore, the phosphorothioate backbone allows a passive diffusion and guarantees the binding to serum proteins and hydrophobic molecules in the cell membrane, reducing the excretion through the kidney [37]. However, once internalized into the endosomes, ASOs must escape in their active form, and this step limits clinical use. Consequently, different strategies are under investigation to target this issue [38,39,40,41].

siRNA delivery is even more challenging since the RNA duplex is highly hydrophilic, making cellular permeability relatively modest. For this reason, several approaches have been tested, including lipids [42,43], peptide transduction domain [44], and cationic polymers [45]; however, the use of nanoparticles seems the most promising, allowing for an enhanced cellular uptake and providing coverage from nucleases [46]. Among these, lipid nanoparticles (LNPs) [46,47] and self-assembled micelle inhibitory RNA (SAMiRNA) are the most studied [48].

If the use of oligonucleotides in conjugation with lipid facilitates the delivery in different districts, it certainly increases the uptake by hepatic cells, in which the presence of LDL receptors allows the entrance of lipid-conjugated oligonucleotides [32]. This limit of the technique became an excellent opportunity for the liver-specific delivery of oligonucleotides, which today are widely studied, as described below. Another promising approach to facilitating liver uptake is the use of a trimer of N-acetylgalactosamine (GalNAc), an amino sugar derivative of galactose that binds asialoglycoprotein receptors on hepatocytes [49]. This approach increases the drug’s stability and potency, reduces the inflammatory response, and is cheaper compared with the other modifications. For all these reasons, since the first experiments with GalNAC as a conjugated molecule for nucleic acid delivery back in the early 1990s [50], an increasing number of studies have been performed; today, several GalNAc-siRNAs conjugate clinical trials are underway for their use as therapeutic agents for a variety of diseases [51].

### 1.4. ASO and siRNA in CVD

ASOs and siRNAs could potentially revolutionize the care of high-risk patients in the cardiovascular field as well, as described below.

**Angiopoietin-like protein 3**. ANGPTL3 plays a central role in the regulation of lipoprotein metabolism in blood plasma, mainly by targeting lipoprotein lipase (LPL) and endothelial lipase (LIPG) [52]. Loss-of-function variants of ANGPTL3 have been found to be associated with decreased levels of lipoproteins in the plasma (LDL and HDL) and triglycerides, with a subsequently decreased risk of coronary artery disease [53,54]. An oligonucleotide targeting ANGPTL3 called AKCEA-ANGPTL3-L_Rx_ (ASO) was tested in a phase 1 clinical trial funded by Ionis Pharmaceuticals (NCT02709850) in which participants were treated with up to 80 mg in a single dose or up to 60 mg weekly. After 6 weeks of treatment, people included in the study had reductions in levels of ANGPTL3 protein, triglycerides, LDL cholesterol (LDL-C), very-low-density lipoprotein cholesterol, non-high-density lipoprotein cholesterol, apolipoprotein B, and apolipoprotein C-III; no serious adverse events occurred [55]. A phase 2 study has also been recently completed in patients with hypertriglyceridemia, type 2 diabetes mellitus, and nonalcoholic fatty liver disease (NCT03371355), achieving the primary endpoint of a significant reduction in triglyceride levels. Additionally, a siRNa named ARO-ANG3 was developed and tested in a phase I/II trial (NCT03747224) in healthy volunteers and dyslipidemic subjects [56]. Subjects received four different single doses compared with placebo. A dose-dependent reduction in triglycerides up to 66% was observed that persisted for at least 16 weeks (time of the study). A non-significant (due to small sample size) dose-dependent reduction in LDL-C was identified. No serious adverse events or dropouts were registered [56]. Two ongoing phase 2 clinical trials are investigating the safety and efficacy of this molecule in participants with homozygous familial hypercholesterolemia (GATEWAY) (NCT05217667) and in adults with mixed dyslipidemia (ARCHES-2) (NCT04832971). The effects brought by drugs targeting ANGPTL3 make them very appealing for the treatment of conditions associated with hypertriglyceridemia. Furthermore, the proven reduction in LDL-c levels brought by AKCEA-ANGPTL3-L_Rx_, which will most likely be confirmed for ARO-ANG3 as well, make them interesting therapies for mixed dyslipidemias.

**Lipoprotein(a).** Lp(a) is an LDL-like lipoprotein, but it contains an ApoA covalently bound to ApoB. Epidemiologic studies showed a connection between elevated levels of Lp(a) and the risk of CVD [57,58], including myocardial infarction [59], coronary disease [60], aortic valve stenosis [61,62], and stroke [63]. High levels of Lp(a) have also been associated with higher all-cause mortality [58].

Different randomized trials investigating therapies that lowered Lp(a) by 20% to 35% failed to provide strong evidence that reducing Lp(a) levels decreases the risk of CHD [7,8,64,65,66,67]. However, this was not the primary endpoint for this trial, and a greater reduction in Lp(a) could be required to produce a significant decrease in the risk of CHD. Lp(a) levels are determined mainly by the LPA gene locus, without significant effects of diet or environment [68]. The mechanisms implicated in the atherogenic development of high Lp(a) levels are not fully elucidated yet, even though prothrombotic and pro-inflammatory contributions have been suggested [69]. PCSK9 inhibition modestly decreased Lp(a) levels [70,71], and statins led to a modest increase [72]. However, there is still a considerable controversy about Lp(a)-lowering therapies [69], with no drugs approved for a specific reduction of Lp(a) plasma levels.

The first study in the field of ASO/siRNA regarding Lp(a) was a randomized, double-blind, placebo-controlled phase 1 study specifically targeting ApoA (European Clinical Trials Database, n. 2012-004909-27) [73]. The study was based on a second-generation ASO, ISIS-APO(a)_Rx_, showing phosphorothioate instead of phosphodiester linkages, and several modified nucleotides including 2′-MOE and 2′-O. In this trial, subjects were randomly assigned to receive either a single-dose or multidose ascending concentrations of ISIS-APO(a)_Rx_ or placebo. The results demonstrated no Lp(a) level reduction at 30 days in the single-dose arm and a dose-dependent decrease in the multi-dose arm. Similar reductions were observed in the numbers of oxidized phospholipids associated with Apo B-100 and Apo(a). The safety and tolerability profile of ISIS-APO(a)_Rx_ supports its role as a potential drug for reducing the risk of CVD and calcific aortic valve stenosis in patients with high Lp(a) levels [73].

A phase 2 clinical trial (NCT02160899) with the aim of assessing the safety, tolerability, pharmacokinetics, and pharmacodynamics of ISIS-APO(a)Rx in subjects with high lipoprotein(a) was performed. Participants were divided into two cohorts (125–437 nmol/L in cohort A; ≥438 nmol/L in cohort B) and randomly assigned to receive either escalating doses of IONIS-APO(a)Rx (100 mg, 200 mg, and then 300 mg once a week for four weeks each) or injections of saline placebo once a week for 12 weeks. At the end of the study (around day 85/99), participants assigned to IONIS-APO(a)Rx had mean Lp(a) reductions of 66.8% (SD 20.6) in cohort A and 71.6% (SD 13.0) in cohort B [74,75].

IONIS-APO(a)L_Rx_, an N-acetyl galactosamine-modified second-generation antisense oligonucleotide, was designed to be highly and selectively taken up by hepatocytes (NCT02414594) [74,75]. Subjects were randomly assigned to receive a single dose of IONIS-APO(a)L_Rx_ of 10–120 mg or multiple doses of 10 mg, 20 mg, or 40 mg subcutaneously. At 30 days from the single-dose administration, meaningful Lp(a) reduction was noticed in all groups. In the multidose groups, Lp(a) reductions of 66% (SD 21.8), 80% (SD 13.7), and 92% (SD 6.5) were observed, respectively, in the 10 mg group, the 20 mg group, and the 4 mg group [75].

More recently, a phase 2 trial tested the efficacy of different dose ranges of IONIS-APO(a)LRx/AKCEA-APO(a)-LRx/TQJ230/Pelacarsen (NCT03070782). These include 20 mg/week, 20 mg/2 weeks, 20 mg/4 weeks, 40 mg/4 weeks, 60 mg/4 weeks, administered through subcutaneous injections showing, respectively, 80%, 58%, 35%, 56%, 72% reductions in Lp(a) levels [76]. Hopefully, these promising data will be confirmed by different ongoing studies, including the HORIZON trial, which aims to enroll more than 7500 patients to assess the effects of 80 mg of IONIS-APO(a)Rx on four cardiovascular diseases endpoints (NCT04023552).

Olpasiran (AMG 890) is an N-acetylgalactosamine (GalNAc)-conjugated (hepatocyte-targeted) siRNA-targeting Lp(a) that was modified with 2′-fluoro and 2′-methoxy substitutions as well as phosphorothioate internucleotide linkages at the termini to stabilize the duplex. It has been tested in a phase 1 placebo-controlled, single-ascending-dose clinical trial (NCT03626662). The primary outcome was met, as single doses were well tolerated by participants. Furthermore, reductions in Lp(a) concentrations of 71–97% has been reported that persisted for several months after the administration of doses of 9 mg or higher [77]. More information is forthcoming once the results from an ongoing phase 2 clinical trial, OCEAN(a)-DOSE, published. This study aims at evaluating the effects of different doses of Olpasiran on the mean percentage change of Lp(a) in subjects with established atherosclerotic disease and Lp(a) > 150nmol/L (NCT04270760) [78].

Another siRNA-targeting Lp(a) is SLN360. A phase 1 clinical trial was conducted to evaluate the safety, tolerability, pharmacokinetics (PK), and pharmacodynamics of this molecule (NCT04606602). Escalating doses were administered in 32 adults with Lp(a) plasma concentrations at screening of approximately ≥ 60 mg/dL without known cardiovascular diseases. A single injection of randomly assigned doses (30 mg, 100 mg, ≤300 mg or ≤600 mg) or placebo were administered and observed for up to 150 days. The main side effects reported were related to injection site, mostly at high doses. SLN360 was shown to reduce Lp(a) levels, in a dose-dependent manner, from 46% to 98%. The reduction persisted for 150 days in up to 81% of subjects [79].

All the aforementioned drugs have been proven to safely reduce Lp(a) levels, even in phase 2 clinical trials, as in the case of IONIS-APO(a)Rx and IONIS-APO(a)L_Rx_. The lack of a clear relationship between Lp(a) levels and CVD make their future in commerce uncertain. Nevertheless, with this new tool, we might be able to clear up this point.

**Apolipoprotein B.** ApoB is a structural protein that constitutes a major component of very-low-density lipoproteins (VLDL), low-density lipoproteins (LDL), and Lp(a); it also works as a ligand for LDL receptors in the liver, where it brings cholesterol-rich particles to be cleared from the bloodstream [80]. Cholesterol-rich, ApoB-containing lipoproteins are considered one of atherosclerotic CVD’s most critical causal agents [81]. A recent study proposed that lowering triglyceride and LDL-C levels might be proportional to the absolute change in ApoB with the following clinical benefit, reducing the CVD risk (i.e., coronary death, myocardial infarction, or coronary revascularization) [82]. Familial hypercholesterolemia (FH) is a disorder primarily caused by extremely high levels of LDL-C; the second-generation ASO mipomersen (commercially named Kynamro) has been suggested as a potential therapeutic drug for FH (NCT00694109) [83]. Mipomersen’s efficacy and safety were tested with one subcutaneous injection per week for 104 weeks; the long-term data showed a prolonged effect in reducing all atherosclerotic lipoproteins: Mipomersen reduced ApoB levels by 31% and LDL-C levels by 28% [83], and the drug was approved by FDA for clinical use. However, the enthusiasm for this drug was curbed by significant side effects such as flu-like symptoms, liver toxicity, and the usual injection site reactions [84]. In particular, hepatic steatosis and hepatic enzyme elevation led to increasing concerns, and mipomersen was withdrawn from the market in 2019. Today, no studies are ongoing on this drug or similar, maybe because of the intrinsic toxic effect on the liver of silencing the ApoB gene.

**Apolipoprotein C III**. ApoC-III is a small glycoprotein secreted by the liver and to a lesser extent in the small intestine that binds to almost all lipoproteins, HDL, LDL, and triglyceride-rich lipoproteins [85]. ApoC-III plays a key role in triglyceride metabolism, impairing the lipolysis of TRLs (triacylglycerol (triglyceride)-rich lipoproteins) by inhibiting lipoprotein lipase and the hepatic uptake of TRLs by remnant receptors [86], which has been found to be associated with coronary artery disease [87]. Loss-of-function mutations in ApoC-III have shown a 40% reduction in triglyceride levels, with a subsequent 40% reduction in CVD [88,89].

ApoC-III is highly abundant in human plasma, so it is difficult to target with a monoclonal antibody, and it has no enzymatic function to inhibit with small chemical drugs; for these reasons, it is an interesting target for the use of ASOs or siRNAs. The first oligonucleotide directly targeting the production of ApoC-III is volanesorsen, an ASO with 2′-MOE modifications and phosphorothioate substitutions, also known as ISIS 304801 or ISIS-APOCIIIRx [90].

Preliminary data about the positive effects of volanesorsen were later confirmed in phase III and phase II clinical trials in patients with hypertriglyceridemia or familial chylomicronemia syndrome (FCS) (NCT01529424; NCT02658175) [91]. In the phase III clinical trial APPROACH, volanesorsen induced an 84% decrease in ApoC-III at 3 months and a subsequent 77% decrease in mean triglyceride levels. Additionally, a 20% increase in ApoB and 46% increase in HDL were observed, while non-HDL cholesterol was reduced by 46% [92,93,94]. No safety issues were reported; however, 45% of the patients treated with volanesorsen displayed thrombocytopenia [94].

The BROADEN study investigates the effect of volanesorsen (formerly IONIS-APOCIIIRx) in participants with familial partial lipodystrophy (NCT02527343). In particular, the study terminated early because sufficient data had accumulated to inform a decision on the further development of volanesorsen. Meanwhile, in May 2019, the European Union approved the use of the updated volanesorsen, renamed Waylivra, in FCS patients with a high risk of pancreatitis and non-responding appropriately to other triglyceride-lowering therapies; the FDA did not approve it yet, mainly waiting for more data regarding the effects on platelet number. Irrespective of these ongoing events, it is known that people with FCS naturally have a highly variable number of platelets, independently of the drug administration.

An upgraded version has since been developed: AKCEA-APOCIII-LRx, also known as ISIS 678354; this oligonucleotide shares the same sequence as volanesorsen, but it is conjugated with GalNAC complex [95]. This small modification, tested in a phase I-II trial, allows for less frequent administration (4 weeks AKCEA-APOCIII-L_Rx_ vs. 1 week ISIS-APOCIIIRx) and leads to an improvement in the atherogenic lipid profile of patients with a favorable safety and tolerability profile (NCT02900027) [95].

The randomized, double-blind, placebo-controlled trial COMPASS has shown how 300 mg of volanesorsen administrated weekly was sufficient to obtain a 73% reduction in triglyceride levels with no serious adverse effect except for reaction at the injection site and no cases of thrombocytopenia [96].

Recently, a siRNA-based therapy against ApoC-III has also been developed:ARO-APOC31001. It was studied in a phase 1 trial (NCT03783377) in healthy volunteers, hypertriglyceridemic patients, and patients with FCS. Preliminary data presented at the AHA meeting in 2019 showed a prolonged reduction in triglyceride levels from 41% to 55% at 16 weeks with no severe adverse effects. Further studies are ongoing, and more time is needed before the approval of ARO-APOC31001 for clinical practice.

Even more than ncRNAs inhibiting ANGPTL3, molecules targeting ApoCIII have been studied for the treatment of patients with hypertrigliceridemia. The modified version of volanesorsen, AKCEA-APOCIII-L_Rx__,_ sounds like a better alternative to its predecessor as it has shown to have fewer side effects and because a less frequent administration is needed. Even if there are only a few studies available, ARO-APOC31001 seems to be a promising molecule as it has already been proven to cause an effective reduction in triglyceride levels.

### 1.5. Inclisiran: A siRNA Directed against PCSK9

Inclisiran, a siRNA directed against PCSK9, is giving very promising results [97]. Preliminary data have described the advantages of using inclisiran, but longer follow-ups are necessary to assess its long-term tolerability, efficacy, and safety [98]. Unlike antibodies that bind the extracellular protein, subtracting it from the interaction with the LDL receptor, inclisiran penetrates hepatocytes blocking PCSK9 mRNA translation; in this way, a single molecule of siRNA is sufficient to reduce the production of several PCSK9 proteins.

It has been established that the key initiating event in atherogenesis is the retention of LDL-C and other cholesterol-rich apolipoproteins-containing lipoproteins within the arterial wall. Clinical trials have indicated that the lower the achieved LDL-C values, the lower the risk of future cardiovascular events, with no lower limit for LDL-C values or the J-curve effect. In addition, studies of the clinical safety of these very low achieved LDL-C values have proved reassuring, albeit monitoring for longer periods is required [99].

In phase I and phase II trials, inclisiran reduced LDL-C by up to 50% in a dose-dependent manner [100,101], as well as non-HDL-C and apolipoprotein B [102].

Two phase III trials have been conducted: one in patients with elevated LDL-C and atherosclerotic CVD (ORION-10 Trial NCT03399370) and another in patients with atherosclerotic CVD risk equivalent (ORION-11 trial NCT03400800) who had elevated LDL-C levels despite receiving statin therapy at the maximum tolerated dose [103,104]. Patients were randomly assigned in a 1:1 ratio to receive either inclisiran (284 mg) or placebo, administered by subcutaneous injection on day 1, on day 90, and every 6 months thereafter over 540 days. The co-primary endpoints in each trial were the placebo-corrected percentage change in LDL-C level from baseline to day 510 and the time-adjusted percentage change in LDL-C level from baseline after day 90 to day 540.

The Orion 10 and Orion 11 studies randomized, respectively, 1561 and 1617 patients who had mean baseline LDL-C levels of 104.7 ± 38.3 mg per deciliter for the former (2.71 ± 0.99 mmol per liter) and 105.5 ± 39.1 mg per deciliter for the latter (2.73 ± 1.01 mmol per liter). Inclisiran at day 510 reduced LDL-C levels by 52.3% (95% confidence interval [CI], 48.8 to 55.7) in ORION-10 and 49.9% (95% CI, 46.6 to 53.1) in ORION-11, with corresponding time-adjusted reductions of 53.8% (95% CI, 51.3 to 56.2) and 49.2% (95% CI, 46.8 to 51.6) (*p* < 0.001 for all comparisons vs. placebo). Adverse events were generally similar in the inclisiran and placebo groups in each study, although injection site adverse events, generally mild and transient, were more frequent with inclisiran than with placebo (2.6% vs. 0.9% in ORION 10 and 4.7% vs. 0.5% in ORION 11). Therefore, Orion 10 and 11 showed that two annual administrations of inclisiran reduced LDL C by approximately 50% [103].

A phase 1 clinical trial was performed in order to assess the pharmacokinetics, pharmacodynamics, safety, and efficacy of inclisiran in subjects with renal impairment (ORION 7) (NCT03159416). In particular, a dose of 300mg of inclisiran was administered to subjects with normal renal function (CrCl of ≥90/mL/min), mild renal impairment (CrCl from 60 to 89 mL/min), moderate renal impairment (CrCl from 30 to 59 mL/min), and severe renal impairment (CrCl from 15 to 29 mL/min). A comparison between the results obtained in this trial and in ORION 1 showed that pharmacodynamic effects and safety were similar in the two groups, leading to the conclusion that no dose adjustment is needed in patients with renal impairment [105]. Similarly, pharmacokinetics, pharmacodynamics, safety and efficacy parameters have been tested in patients with mild (Child–Pugh A) or moderate (Child–Pugh B) hepatic impairment, as compared with subjects with normal liver function in the ORION 6 trial. Those results indicated an up to twofold increase in pharmacokinetic exposure in subjects with moderate hepatic impairment, while the pharmacodynamic properties were unchanged. Inclisiran was considered safe in these subjects, and no dose adjustment was needed [106].

In a few years, the results of the cardiovascular outcome trial (ORION 4, NCT03705234) and the Victorion initiate trial (NCT04929249) will be available. The former, if confirmed, will demonstrate how two doses of inclisiran per year would be sufficient to ensure CVD risk reduction comparable with other drugs that instead require more frequent dosing (daily or weekly). The latter, on the other hand, appraising the effectiveness of an inclisiran-first implementation strategy (added to the maximum tolerated dose of statins) in subjects with atherosclerotic cardiovascular disease who are not able to reach their LDL goal, compared with usual care.

This drug has given incredible results in terms of safety and efficacy. Furthermore, the twice-a-year administration makes it game changing for patients suffering with ASCVD, as it increases compliance.

The inclusion/exclusion criteria and data regarding the available results of the trials are present in Supplementary Table S1. Details on the genes associated with the different target proteins are available in Supplementary Table S2. Table 2 presents the main molecules discussed with the main effects on LDL, TG and Lp(a).

## 2. Discussion

### ASOs vs. siRNAs: Who Is Getting the Upper Hand?

It is difficult to establish which is better between ASOs and siRNA. They have several differences, especially at the structural level: The former is a single-stranded ncRNA, while the latter is a double-stranded ncRNA. This feature confers on siRNAs a greater stability, making it more appealing for in vitro studies as compared with ASOs. Nevertheless, this advantage is not necessarily true for in vivo studies and clinical purposes. Conversely, the presence of two strands of RNA may bring a higher off-target effect in siRNA-based drugs since both the sense and antisense strands could be active.

Even though they are structurally different, they share the same mechanism of action or rationale: RNA interference, which consists of impairing the production of a specific protein by blocking/degrading its mRNA and hence inhibiting its synthesis. Different chemical modifications are available to improve the pharmacodynamic as well as pharmacokinetic characteristics of this molecules. Accordingly, the potency and stability of the molecule and the efficacy of the drug need to be evaluated independently for every single compound [107].

Both siRNA and ASO-based therapies have been involved in cardiovascular medicine. In this context, the first siRNA-based therapy approved by FDA is patisiran, for patients with transthyretin-mediated amyloidosis [49,51]. Furthermore, for lipid-lowering therapy, a particular siRNAs is revolutionizing the therapeutical approach to hypercholesterolemia: inclisiran, which achieves significant reduction in PCSK9 expression and subsequently in LDL levels with just two doses per year [103].

ASO was also suggested for the treatment of cardiovascular diseases such as heart failure (HF). Since defective cardiac Ca^2+^ homeostasis is crucial in HF development, it was suggested to use an antisense oligonucleotide to address phospholamban, a key regulator of Ca^2+^ equilibrium in hypertrophied and failing cardiomyocytes [108].

A tremendous number of works on non-coding RNA have been published, unraveling the mechanisms underlying the world of microRNA and long non-coding RNA, in different medical fields including cardiovascular [109,110]. A recent case report has shown findings for a 6-year-old girl with a rare genetic disease who, once the culprit DNA mutation was identified, was treated with a specific oligonucleotide antisense (milasen, a 22-nucleotide antisense oligonucleotide with phosphorothioate in the backbone and 2′-O-MEO sugar) [111]. This study serves as proof of the concept of the possible use of ASOs or siRNAs in clinical practice for custom therapies as well; the system is indeed flexible, quick, and effective. It also paves the way for the use of oligonucleotides for other therapeutic approaches like gene replacement [112] or genome editing with CRISPR/Cas9 [113].

Overall, early experience with using RNA-based gene-silencing provides several advantages including great specificity and a low frequency of drug administration. However, some aspects associated with the use of RNA-based drugs still need to be improved, like the need for increased stability, the reduction in off-target effects, and the advent of flulike symptoms. Even if preliminary studies showed overall reassuring results in terms of safety, there are still some unsolved issues (i.e., the platelet reduction observed for revusiran) [48,49].

The costs related to the production of oligonucleotides and their conjugates and to their reimbursement once the molecules are available on the market will require a significant economic investment. Nevertheless, it is important to put these costs in the right perspective as the costs of these highly effective, life-changing medications may outweigh the costs of caring for patients left untreated [114].

Although we have witnessed a few failures in the RNA-based therapies field, these technologies remain very promising, and increasing numbers of companies are investing in the study of these molecules. The establishment of new drug families requires a long time; beyond the positive results, it is fundamental to build confidence among clinicians. First steps have already been taken, and even today, several companies are sponsoring clinical trials of antisense oligonucleotides.

These data are very encouraging; however, further studies are needed to introduce those new molecules into clinical practice.

## Figures and Tables

**Table 1:** Characteristics of Different Anti-hypercholesterolemia Agents.

	Small Molecules	Antibodies	Antisense Oligonucleotides (ASO)	Short Interfering RNA (siRNA)
**Structure**	Organic compound	Protein	Single-stranded RNA	Double-stranded RNA
**Mass (kDa)**	<1	~150	~12	~21
**Mechanism of action**	Blocks enzyme or receptor	Blocks protein	Blocks gene mRNA transcripts	Blocks gene mRNA transcripts
**Potential for off-target adverse effects**	High	Low	Low	Low
**Immunogenicity**	Low	High	High	High
**Efficacy**	50% reduction in LDL	60% reduction in LDL	90% reduction in Lp(a)	50% reduction in LDL
**Drug response variability**	High	High	Low	Low
**Half-life**	Days	Weeks	Months	>1 year
**Administration route**	Oral	Subcutaneous	Subcutaneous	Subcutaneous
**Dosing frequency**	Daily	Weekly to twice monthly	Monthly	Twice yearly

**Table 2:** The main effects of the overviewed molecules and their targets on LDL, TG, and Lp(a).

Target Protein	Molecule (Type)	LDL-C	TG	Lp(a)
ANGPTL 3	AKCEA-ANGPTL3-L_RX_ (ASO)	+	+	
ANGPTL 3	ARO-ANG3 (SiRNA)		+	
Lipoprotein(a)	ISIS-APO(a)_Rx_ (ASO)			+
Lipoprotein(a)	IONIS-APO (a) L_Rx_ (ASO)			+
Lipoprotein(a)	Olpasiran (siRNA)			+
Lipoprotein(a)	SLN360 (siRNA)			+
Apolipoprotein B	Mipomersen (ASO)	+	+	+
Apolipoprotein C III	Volanesorsen (ASO)		+	
Apolipoprotein C III	AKCEA-APOCIII-L_RX_ (ASO)		+	
Apolipoprotein C III	ARO-APOC31001 (siRNA)		+	
PCSK9	Inclisiran (siRNA)	+		

## Supplementary Materials

The following supporting information can be downloaded at: https://www.mdpi.com/article/10.3390/jcm11133884/s1, Table S1: Inclusion and exclusion criteria; Table S2: Genes associated with the different target proteins.
