# Supplementary material for: Antisense Oligonucleotides and Small Interfering RNA for the Treatment of Dyslipidemias
Source: J Clin Med. 2022 Jul 4;11(13):3884. doi: 10.3390/jcm11133884 (PMC9267663; doi:10.3390/jcm11133884)
Supplement: Supplementary file 1 [file jcm-11-03884-s001.zip › jcm-1693738-supplementary.pdf]

# Supplementary files

**Supplementary Table S1**

| Name | Trial | Type of study | Inclusion / Exclusion criteria | Baseline value | Post value | % change |
|---|---|---|---|---|---|---|
| AKCEA-ANGPTL3-LRx/ IONIS-ANGPTL3-LRx/ Vupanorsen | NCT02709850 | Phase 1 clinical trial | Inclusion Criteria<br>1. Must have given written informed consent (signed and dated) and any authorizations required by local law and can comply with all study requirements<br>2. Healthy males or females aged 18 to 65 years, inclusive at the time of informed consent<br>3. Body mass index (BMI) ≤ 35.0 kg/m2<br>4. Fasting triglycerides between 90 and 150 mg/dL at Screening for the 40 and 80 mg SAD cohorts, and fasting triglycerides ≥ 150 mg/dL for all other cohorts (up to 1 rescreen allowed for triglycerides; lower triglycerides levels may be allowed upon consultation with the Sponsor).<br>5. Fasting LDL-C > 70 mg/dL at Screening<br>6. Females must be non-pregnant and non-lactating, and | Single dose group baseline characteristics:<br><br>Dose: Placebo ;20 mg; 40 mg; 80 mg Characteristic (N=3) (N=3) (N=3) (N=3) Age (years, Range) 59 (58-60) 52 (44-62) 62 (59-64) 46(44-48) Gender (M:F) 1:2 3:0 1:2 3:0 BMI (kg/m2) 27.1 (3.7) 28.1 (0.5) 29.0 (4.2) 26.5 (3.9) ANGPTL3, ng/ml (plasma) 149.4 (70.6) 72.6 (9.6) 130.9 (39.3) 119.4 (17.7) | Single dose group characteristics at 15 days after the administration: Dose: Placebo; 20 mg IONIS-ANGPTL3-LRx; 40 mg IONIS-ANGPTL3-LRx; 80 mg IONIS-ANGPTL3-LRx Characteristic (N=3) (N=3) (N=3) (N=3) ANGPTL3, ng/mL 156.1 (36.4) 102.5 (2.9) 103.1 (41.2) 49.7 (8.9) Triglycerides, mg/dL 364 | |

| | | | | | | |
|---|---|---|---|---|---|---|
| | | | either surgically sterile (e.g., tubal occlusion, hysterectomy, bilateral salpingectomy, bilateral oophorectomy) or postmenopausal (defined as 12 months of spontaneous amenorrhea without an alternative medical cause and follicle-stimulating hormone (FSH) levels in the postmenopausal range for the laboratory involved)<br>7. Males must be surgically sterile, abstinent or, if engaged in sexual relations with a female of childbearing potential, the subject must be using an acceptable contraceptive method from the time of signing the informed consent form until at least 30 days (single-dose cohorts) or 13 weeks (multiple-dose cohorts) after the last dose of Study Drug (ISIS 703802 or placebo)<br><br>Exclusion Criteria<br>1. Clinically-significant abnormalities in medical history including acute coronary syndrome, major surgery within 3 months of screening, planned surgery that would occur during the study or physical examination or | Triglycerides, mg/dL 113 (25) 231 (41) 132 (14) 137 (36)<br>LDL-C, mg/dL 116 (8) 140 (18) 162 (10) 128 (12)<br>VLDL-C, mg/dL 24 (6) 51 (6) 25 (4) 29 (7)<br>ApoB, mg/dL ND ND ND ND<br>Non-HDL-C, mg/dL 142 (3) 184 (17) 185 (18) 158 (14)<br>Total Cholesterol, mg/dL 189 (8) 216 (21) 239 (11) 217 (13)<br>HDL-C, mg/dL 48 (7) 32 (5) 50 (9) 59 (4)<br>///<br>Multiple administration dose (1 dose/week for 6 weeks) baseline characteristics : Dose: Placebo (N=8); 10 mg (N=6) ; 20 mg (N=6); 40 mg | (443) 190 (34) 107 (32) 59 (14)<br>LDL-C, mg/dL 116 (31) 154 (27) 146 (18) 97 (7)<br>VLDL-C, mg/dL 78 (98) 38 (7) 21 (7) 12 (3)<br>Non-HDL-C, mg/dL 194 (68) 192 (20) 167 (20) 109 (4)<br>Total Cholesterol, mg/dL 231 (58) 228 (27) 218 (20) 163 (6)<br>HDL-C, mg/dL 37 (10) 36 (8) 51 (17) 54 (6)<br>////<br>Multiple administration dose (1 dose/week for 6 weeks) at day 43 after the initiation of the drug: | |

| | | | | | | |
|---|---|---|---|---|---|---|
| | | | other screening results such as ECGs at Screening<br>2. Clinically-significant abnormalities in the following screening laboratory values including the following: • Urine protein/creatinine (P/C) ratio ≥ 0.2 mg/mg. In the event of a P/C ratio above this threshold, eligibility may be confirmed by a quantitative total urine protein measurement of < 150 mg/24-hour • Positive test (including trace) for blood upon urinalysis. In the event of a positive test, eligibility may be confirmed with a urine microscopy showing < 5 red blood cells (RBCs) per high power field • Alanine aminotransferase (ALT), aspartate aminotransferase (AST), bilirubin, alkaline phosphatase, serum creatinine, blood urea nitrogen (BUN) > upper limit of normal (ULN) • Platelet count < lower limit of normal (LLN) • Show evidence of uncorrected hypothyroidism or hyperthyroidism hormone results at Screening. Subjects receiving dose-stable thyroid replacement therapy for at least 3 months prior to screening will be allowed to participate if thyroid tests (thyroid-stimulating hormone | (N=6) ;60 mg (N=6)<br>N. patient/each dose<br>Mean age (range) — yr 55 (46–64) 46 (28–64) 52 (28–65) 54 (39–62) 56 (49–62)<br>Sex ratio (male:female) 6:2 5:1 6:0 6:0 6:0<br>BMI† 28.0±3.8 27.9±3.4 27.0±4.0 26.4±3.2 28.5±2.6<br>ANGPTL3 — ng/ml 126.1±32.5 84.5±23.5 96.8±19.3 112.4±7.8 109.7±38.3<br>Triglycerides — mg/dl 201±36 212±107 196±50 212±62 168±60<br>LDL cholesterol — mg/dl 132±16 133±41 141±29 154±32 128±42 | Dose: Placebo (N=8) ; 10 mg (N=6); 20 mg (N=5); 40 mg (N=6) ;60 mg (N=6)<br>ANGPTL3 — ng/ml :132.5±38.9 45.3±22.9† 24.5±7.5† 21.1±5.0† 16.6±8.1†<br>Triglycerides — mg/dl :183±76 135±55 73±20† 93±24‡ 82±27†<br>LDL cholesterol — mg/dl : 151±18 126±29 124±24 115±31‡ 85±26†<br>VLDL cholesterol — mg/dl 37±15 27±11 15±4† 19±5‡ 16±6†<br>Apolipoprotein B — mg/dl 122±19 102±22 | |

| | | | | | | | |
|---|---|---|---|---|---|---|---|
| | | | [TSH]/T3/T4) show that subject is euthyroid<br>3.Active infection requiring systemic antiviral or antimicrobial therapy that will not be completed prior to the first day Study Drug product is administered to the subject (Day 1)<br>4. Unwillingness to comply with study procedures, including follow-up, as specified by this protocol, or unwillingness to cooperate fully with the Investigator 5. Known history or positive test for human immunodeficiency virus (HIV), hepatitis C, or chronic hepatitis B<br>6. Malignancy within 5 years, except for basal or squamous cell carcinoma of the skin or carcinoma in situ of the cervix that has been successfully treated<br>7. Treatment with another investigational drug, biological agent, or device within 4 weeks of screening or 5 half-lives of investigational agent, whichever is longer<br>8. Treatment with any non-Ionis oligonucleotide (including small interfering RNA [siRNA]) at any time or prior treatment with an Ionis oligonucleotide or siRNA | VLDL cholesterol — mg/dl 38±10 39±20 38±12 38±11 33±13 Apolipoprotein B100 — mg/dl 108±13 107±28 120±15 120±13 103±39 Non-HDL cholesterol — mg/dl 170±19 172±36 180±30 192±27 161±50 Total cholesterol — mg/dl 216±12 217±35 216±32 230±31 206±48 HDL cholesterol — mg/dl 46±11 46±16 37±7 38±5 45±10 Apolipoprotein AI — mg/dl 146±18 149±32 131±15 129±10 137±16 Apolipoprotein C-III — mg/dl 12.6±2.9 11.2±4.6 9.7±1.9 11.0±2.8 9.7±3.1 | 99±13‡ 90±19‡ 78±22† Non-HDL cholesterol — mg/dl 188±25 153±28‡ 139±26‡ 133±32‡ 101±31† Total cholesterol — mg/dl 230±20 197±27‡ 171±30† 168±33† 134±29† HDL cholesterol — mg/dl 42±12 44±16 32±5 35±4 33±10 Apolipoprotein AI — mg/dl 146±15 143±36 115±15† 112±13† 105±23† Apolipoprotein C-III — mg/dl 12.8±3.2 9.1±3.8 4.2±2.3† 5.7±3.1† 3.8±1.0† | |

| | | | | | | |
|---|---|---|---|---|---|---|
| | | | within 9 months of screening. Subjects who have previously received only a single dose of an Ionis oligonucleotide as part of a clinical study may be included if ≥ 4 months have elapsed since dosing<br>9. History of bleeding diathesis or coagulopathy<br>10. Regular use of alcohol within 6 months prior to screening (> 7 drinks/week for females, > 14 drinks/week for males: 1 drink = 5 ounces [150 mL] of wine or 12 ounces [360 mL] of beer or 1.5 ounces [45 mL] of hard liquor); or use of soft drugs (such as marijuana) within 3 months prior to screening, or hard drugs (such as cocaine or phencyclidine [PCP]) within 1-year prior to screening, or positive urine drug result at Screening<br>11. Use of concomitant drugs (including herbal or over-the-counter [OTC] medications) unless authorized by the Sponsor Medical Monitor<br>12. Known contraindication and/or allergy to heparin. Subjects who meet this criterion may be included in the study with the Sponsor's approval as the heparin assessments can be optional for a subset of subjects | Lipoprotein(a) — nmol/liter 35±23 70±72 32±53 20±28 10±14 | Lipoprotein(a) — nmol/liter 32±21 71±69 13±12 18±24 5±8†<br>† The difference in comparison with the placebo group was significant (P<0.01).<br>‡ The difference in comparison with the placebo group was significant (P<0.05). | |

| | | | | | | |
|---|---|---|---|---|---|---|
| | | | 13. Smoking > 10 cigarettes/day; also, subjects with a significant change in smoking habits within 1-month prior to screening should be excluded<br>14. Blood donation of 50 to 499 mL within 30 days of screening or of > 499 mL within 60 days of screening<br>15. Have any other conditions that, in the opinion of the Investigator, would make the subject unsuitable for inclusion, or could interfere with the subject participating in or completing the study | | | |
| AKCEA-ANGPTL3-LRx/ IONIS-ANGPTL3-LRx/ Vupanorsen | NCT 03371355 | Phase 2 | Key Inclusion Criteria:<br>• Plasma triglycerides (TG) at Screening greater than (>)150 milligrams per deciliter (mg/dL) and at qualification of >150 mg/dL.<br>• Documented history of hepatic steatosis with baseline magnetic resonance imaging (MRI) indicating hepatic fat fraction (HFF) greater than (>) 8%. | | | TC<br>Pooled placebo: -4.8 (-15.19 to 5.51)<br>Cohort B ( 40 mg): -21.3 (-32.35 to -10.31)<br>Cohort C ( 80mg): -41.9 (-52.88 to -30.89)<br>Cohort A ( 20 mg): -36.5 (-49.38 to -23.58)<br><br>LDL-C<br>Pooled placebo: -2.5 (-11.21 to 6.14)<br>Cohort B ( 40 mg): 5.0 (-4.78 to 14.70) |

| | | | | | | |
|---|---|---|---|---|---|---|
| | | | <ul><li>Diagnosis of Type 2 diabetes mellitus with hemoglobin A1c (HbA1c) >6.5 and less than or equal to (≤) 10% at Screening.</li><li>Must have been on a stable dose of oral antidiabetic therapy for a minimum of 3 months prior to Screening.</li><li>Body mass index between 27- 40 kilograms per meter square (kg/m^2), inclusive, at Screening.</li></ul>Key Exclusion Criteria:<ul><li>Type 1 diabetes mellitus.</li><li>Active chronic liver disease, alcoholic liver disease, Wilson's disease hemochromatosis, primary biliary cirrhosis, primary sclerosing cholangitis, genetic hemochromatosis, known or suspected hepatocellular carcinoma, history of or planned liver transplant for end-stage liver disease of any etiology.</li></ul> | | | Cohort C ( 80mg): -11.1 (-20.38 to -1.90)<br>Cohort A ( 20 mg): -11.3 (-21.76 to -0.84)<br><br>HDL-C<br>Pooled placebo: 2.2 (-0.38 to 4.69)<br>Cohort B ( 40 mg): -0.6 (-3.25 to 2.14)<br>Cohort C ( 80mg): -6.4 (-9.13 to -3.77)<br>Cohort A ( 20 mg): -2.0 (-5.11 to 1.12)<br><br>VLDL-C<br>Pooled placebo: -6.5 (-11.64 to -1.34)<br>Cohort B ( 40 mg): -15.6 (-21.47 to -9.78)<br>Cohort C ( 80mg): -21.9 (-27.41 to -16.36)<br>Cohort A ( 20 mg): -17.3 (-23.48 to -11.13)<br><br>Non-HDL-C<br>Pooled placebo: -7.0 (-17.45 to 3.44)<br>Cohort B ( 40 mg): -20.9 (-32.01 to -9.70) |

| | | | | | | |
|---|---|---|---|---|---|---|
| | | | • Documented history of advanced liver fibrosis.<br>• History of cirrhosis and/or hepatic decompensation including ascites, hepatic encephalopathy, or variceal bleeding.<br>• History of clinically significant acute cardiac event within 6 months before Screening.<br>• History of heart failure with New York Heart Association (NYHA) greater than Class II.<br>• Use of Insulin or insulin analogs, glucagon-like peptide-1 (GLP-1) agonists, and peroxisome proliferator-activated receptor gamma (PPARγ) agonists (pioglitazone or rosiglitazone).<br>• Weight change >5% within 3 months before Screening.<br>• Conditions contraindicated for magnetic resonance imaging (MRI) | | | Cohort C ( 80mg): -35.4 (-46.54 to -24.33)<br>Cohort A ( 20 mg): -34.4 (-47.39 to -21.34)<br><br>ApoB<br>Pooled placebo: -3.63 (-9.98 to 2.71)<br>Cohort B ( 40 mg): -6.08 (-12.82 to 0.66)<br>Cohort C ( 80mg): -13.46 (-20.20 to -6.72)<br>Cohort A ( 20 mg): -8.91 (-16.91 to -0.91)<br><br>ApoB: ApoB-48<br><br>Pooled placebo: -0.139 (-0.90 to 0.62)<br>Cohort B ( 40 mg): -1.140 (-1.93 to -0.35)<br>Cohort C ( 80mg): -1.942 (-2.75 to -1.14)<br>Cohort A ( 20 mg): -1.230 (-2.16 to -0.30)<br><br>ApoB: ApoB-100 |

| | | | | | | | |
|---|---|---|---|---|---|---|---|
| | | | | procedures including any metal implant (example, heart pacemaker, rods, screws, aneurysm clips). | | | Pooled placebo: -3.757 (-9.94 to 2.43)<br>Cohort B ( 40 mg): -4.643 (-11.21 to 1.92)<br>Cohort C ( 80mg): -11.346 (-17.91 to -4.78)<br>Cohort A ( 20 mg): -7.174 (-14.97 to 0.62)<br>ApoCIII<br>Pooled placebo: -0.729 (-2.58 to 1.12)<br>Cohort B ( 40 mg): -5.482 (-7.42 to -3.54)<br>Cohort C ( 80mg): -9.228 (-11.18 to -7.28)<br>Cohort A ( 20 mg): -7.588 (-9.86 to -5.32)<br><br>ApoA1<br>Pooled placebo: 5.0 (-1.11 to 11.15)<br>Cohort B ( 40 mg): -11.8 (-18.32 to -5.30)<br>Cohort C ( 80mg): -30.1 (-36.63 to -23.65) |

| | | | | | | |
|---|---|---|---|---|---|---|
| | | | | | | Cohort A ( 20 mg): -16.1 (-23.68 to - 8.57) |
| ARO-ANG3 | NCT03747224 | Phase 1 | Inclusion Criteria:<br>• Women of child bearing potential must have a negative pregnancy test, cannot be breastfeeding and must be willing to use contraception<br>• Willing to provide written informed consent and to comply with study requirements<br>• On a stable diet for at least 4 weeks with no plans to significantly alter diet or weight over course of study<br>• Normal electrocardiogram (ECG) at Screening<br>Exclusion Criteria:<br>• Clinically significant health concerns<br>• Regular use of alcohol within one month prior to Screening<br>• Use of an investigational agent or | N/A | N/A | N/A |

| | | | | | | |
|---|---|---|---|---|---|---|
| | | | <ul><li>device within 30 days prior to dosing or current participation in an investigational study</li><li>Recent use of illicit drugs</li><li>Use of more than two tobacco/nicotine containing or cannabis products per month within 6 months prior to drug administration (applicable only to Normal Healthy Volunteers)</li></ul> | | | |
| ARO-ANG3 | NCT05217667 | Phase 2 | Inclusion Criteria:<ul><li>Fasting LDL-C >100 mg/dL at Screening</li><li>Weight of ≥ 30 kg and body mass index ≥ 16 and ≤ 40 kg/m2</li><li>Diagnosis of HoFH based on a supportive genetic test</li><li>On stable maximally tolerated lipid lowering therapy</li><li>Willing to abide by stable low-fat, low-cholesterol, heart-healthy diet for at least 4 weeks prior to Day 1</li></ul> | Still ongoing | Still ongoing | Still ongoing |

| | | | | | | |
|---|---|---|---|---|---|---|
| | | | • Participants of childbearing potential (males & females) must agree to use highly-effective contraception during the study and for at least 24 weeks from the last dose of study medication.<br>• Women of childbearing potential must have a negative pregnancy test and cannot be breastfeeding<br>• Women of childbearing potential on hormonal contraceptives must be stable on the medications for ≥ 2 menstrual cycles prior to Day 1<br>• Willing to provide written informed consent and to comply with study requirements<br>Exclusion Criteria:<br>• Current use or use within 365 days from Day 1 of any hepatocyte targeted small interfering RNA oligonucleotides (siRNA) or antisense | | | |

| | | | | | | |
|---|---|---|---|---|---|---|
| | | | oligonucleoside molecule<br>• Use of evinacumab (some exceptions apply)<br>• Fasting TG > 300 mg/dL at Screening<br>• Presence of any clinically significant uncontrolled endocrine disease known to influence serum lipids or lipoproteins<br>• Newly diagnosed (within 3 months prior to informed consent) or poorly controlled diabetes (Hemoglobin A1c > 9%)<br>• Use of systemic corticosteroids (some exceptions apply)<br>• Symptoms of myocardial ischemia or severe left ventricular dysfunction<br>• History of malignancy within 3 years of Day 1 (some exceptions apply)<br>• Planned cardiac procedure/surgery such as coronary artery bypass graft (CABG) surgery, percutaneous | | | |

| | | | | | | |
|---|---|---|---|---|---|---|
| | | | coronary intervention (PCI), carotid surgery or stenting, or carotid revascularization<br>Note: additional inclusion/exclusion criteria may apply per protocol | | | |
| ARO-ANG3 | NCT04832971 | Phase 2 | Inclusion Criteria:<br>• Based on medical history, evidence of TG ≥ 150 mg/dL but ≤ 499 mg/dL on more than one occasion<br>• Fasting levels at Screening of LDL-C ≥ 70 mg/dL OR non-HDL-C ≥ 100 mg/dL after at least 4 weeks of stable optimal statin therapy<br>• Mean fasting TG ≥ 150 mg/dL and ≤ 499 mg/dL during Screening collected at two separate and consecutive visits and at least 7 days apart and not more than 14 days apart<br>• Willing to follow diet counseling and maintain a stable diet per Investigator | Still ongoing | Still ongoing | Still ongoing |

| | | | | | | |
|---|---|---|---|---|---|---|
| | | | judgment based on local standard of care<br>• Women of childbearing potential on must have a negative pregnancy test, cannot be breastfeeding, and must be willing to use contraception<br>• Women of childbearing potential on hormonal contraceptives must be stable on the medication for ≥ 2 menstrual cycles prior to Day 1<br>• Willing to provide written informed consent and to comply with study requirements<br>Exclusion Criteria:<br>• Current use or use within 365 days from Day 1 of any hepatocyte targeted siRNA or antisense oligonucleotide molecule<br>• Active pancreatitis within 12 weeks prior to Day 1<br>• Any planned bariatric surgery or similar | | | |

| | | | | | | |
|---|---|---|---|---|---|---|
| | | | procedures to induce weight loss from consent to end of study<br>• Acute coronary syndrome event within 24 weeks of Day 1<br>• Major surgery within 12 weeks of Day 1<br>• Planned coronary intervention (e.g., stent placement or heart bypass) during the study<br>• Uncontrolled hypertension<br>• Human immunodeficiency virus (HIV) infection, seropositive for Hepatitis B (HBV), seropositive for Hepatitis C (HCV)<br>• Uncontrolled hypothyroidism or hyperthyroidism<br>• Hemorrhagic stroke within 24 weeks of Day 1<br>• History of bleeding diathesis or coagulopathy<br>• Current diagnosis of nephrotic syndrome | | | |

| | | | | | | |
|---|---|---|---|---|---|---|
| | | | • Systemic use of corticosteroids or anabolic steroids within 4 weeks prior to Day 1 or planned use during the study<br>• Malignancy within the last 2 years prior to date of consent requiring systemic treatment (some exceptions apply) | | | |
| ISIS-APO(a)$_{Rx}$ | European Clinical Trials Database, n. 2012-004909-27 | Phase 1 | healthy adults, aged 18–65 years, with body-mass index (BMI) less than 32·0 kg/m² and Lp(a) concentration of 25 nmol/L (100 mg/L) or more. To be included, women had to not be pregnant and not lactating and either surgically sterile (eg, tubal occlusion, hysterectomy, bilateral salpingectomy, bilateral oophorectomy) or postmenopausal, whereas men had to be surgically sterile, abstinent, or, if engaged in sexual relations of childbearing potential, must have been using an acceptable contraceptive method during treatment and for at least 30 days (single-dose groups) or 12 weeks (multi-dose groups) after the last dose. Further exclusion | | | Total cholesterol ; LDL cholesterol, ApoB; HDL-cholesterol DID NOT DIFFER SIGNIFICANTLY.<br><br>Multi-dose cohorts: Dose-dependent decreases in Lp(a) from baseline to day 36 (100 mg 39ɫ6%, p=0ɫ005 vs placebo; 200 mg 59ɫ0%, p=0ɫ001 vs placebo; 300 mg 77ɫ8%, p=0ɫ001 vs placebo;<br><br>Dose-dependent, mean percent decreases in OxPL-apoB from baseline to day 36 in OxPL- |

| | | | | | | |
|---|---|---|---|---|---|---|
| | | | criteria included clinically signifi cant abnormalities in medical history or laboratory test results, infection requiring systemic antiviral or antimicrobial therapy, known history of or positive test for HIV, hepatitis C, or chronic hepatitis B, malignancy, treatment with any other drug or non-ISIS oligonucleotide, heavy smoking, and high alcohol consumption. | | | apoB (100 mg 26ł1%, p=0ł020 vs placebo; 200 mg 55ł1%, p OxPL-apo(a) from baseline to day 36 (200 mg 38ł1%, p=0ł020 vs placebo; 300 mg 84ł2%, p=0ł001 vs placebo;<br>Single doses : (50–400 mg) did not decrease Lp(a) concentrations at day 30. |
| ISIS-APO(a)Rx | NCT02160899 | Phase 2 | Inclusion Criteria:<br>• Males or females aged 18-65 inclusive<br>• Females must be non-pregnant and non-lactating, and either surgically sterile (e.g., tubal occlusion, hysterectomy, bilateral salpingectomy, bilateral oophorectomy) or post-menopausal (defined as 12 months of spontaneous amenorrhea without an alternative medical cause and follicle-stimulating hormone | | | Cohort A: Placebo- Hide Arm/Group Description: Participants received placebo (normal saline) subcutaneously on Days 1, 8, 15, 22, 29, 36, 43, 50, 57, 64, 71, and 78.<br>Mean (Standard Deviation) variation of Lp(a): -3.7 (13.7)<br>Cohort A: ISIS-APO(a)Rx < 2000 mg- Hide Arm/Group Description: Participants |

| | | | | | | |
|---|---|---|---|---|---|---|
| | | | (FSH) levels in the postmenopausal range for the laboratory involved)<br>• Males must be surgically sterile, abstinent or if engaged in sexual relations with a female of child-bearing potential, the participant must be using an acceptable contraceptive method from the time of signing the informed consent form until at least 16 weeks after the last dose of Study Drug<br>• Body mass index (BMI) ≤40 kg/m2<br>• Lipoprotein(a) ≥50 and <175 mg/dL at time of screening (Cohort A)<br>• Lipoprotein(a) ≥175 mg/dL at time of screening (Cohort B)<br>Exclusion Criteria:<br>• Clinically significant abnormalities in medical history (e.g., documented previous myocardial infarction, percutaneous coronary intervention (PCI), or | | | received ISIS-APO(a)Rx subcutaneously: 100 mg on Days 1, 8, 15, and 22; 200 mg on Days 29, 36, 43, and 50 unless down-titrated; and 300 mg on Days 57, 64, 71, and 78 unless down-titrated. Mean (Standard Deviation) variation of Lp(a): -44.5 (13.1)<br><br>Cohort A: ISIS-APO(a)Rx >= 2000 mg- Hide Arm/Group Description: Participants received ISIS-APO(a)Rx subcutaneously: 100 mg on Days 1, 8, 15, and 22; 200 mg on Days 29, 36, 43, and 50 unless down-titrated; and 300 mg on Days 57, 64, 71, and 78 unless down-titrated. Mean (Standard Deviation) variation |

| | | | | | | |
|---|---|---|---|---|---|---|
| | | | major surgery within 3 months of screening, planned surgery that would occur during the study) or physical examination at screening<br>• Clinically significant abnormalities in screening laboratory values that would render a participant unsuitable for inclusion<br>• Active infection requiring systemic antiviral or antimicrobial therapy that will not be completed prior to Study Day 1<br>• Known history or positive test for human immunodeficiency virus (HIV), hepatitis C, or chronic hepatitis B<br>• Malignancy within 5 years, except for basal or squamous cell carcinoma of the skin or carcinoma in situ of the cervix that has been successfully treated | | | of Lp(a): -70.0 (19.6)<br><br>Cohort B: Placebo - Hide Arm/Group Description: Participants received placebo (normal saline) subcutaneously on Days 1, 8, 15, 22, 29, 36, 43, 50, 57, 64, 71, and 78. Mean (Standard Deviation) variation of Lp(a): -5.6 (3.9)<br><br>Cohort B: ISIS-APO(a)Rx < 2000 mg - Hide Arm/Group Description: Participants received ISIS-APO(a)Rx subcutaneously: 100 mg on Days 1, 8, 15, and 22; 200 mg on Days 29, 36, 43, and 50 unless down-titrated; and 300 mg on Days 57, 64, 71, and 78 unless down-titrated. Mean (Standard |

| | | | | | | |
|---|---|---|---|---|---|---|
| | | | • History of bleeding diathesis or coagulopathy<br>• Recent history of, or current drug or alcohol abuse<br>• Participant with Lp(a) ≥50 and <175 mg/dL may not receive concomitant niacin therapy during the period 8 weeks prior to screening through the end of the Post-Treatment Evaluation Period<br>• Use of statins, ezetimibe or fibrates unless on a stable regimen for at least 8 weeks prior to dosing and will remain on a stable regimen for the duration of the study<br>• Use of lipid or Lp(a)-specific apheresis within 4 weeks prior to Screening through the end of the Post-Treatment Evaluation Period<br>• Use of concomitant drugs (including herbal or over-the-counter | | | Deviation) variation of Lp(a):<br><br>Cohort B: ISIS-APO(a)Rx >= 2000 mg- Hide Arm/Group Description: Participants received ISIS-APO(a)Rx subcutaneously: 100 mg on Days 1, 8, 15, and 22; 200 mg on Days 29, 36, 43, and 50 unless down-titrated; and 300 mg on Days 57, 64, 71, and 78 unless down-titrated. Mean (Standard Deviation) variation of Lp(a): -71.6 (13.0) |

| | | | | | | |
|---|---|---|---|---|---|---|
| | | | (OTC) medications other than ibuprofen, Benadryl or topical steroids) unless authorized by the Sponsor Medical Monitor<br>• Blood donation of 50-499 mL within 30 days of screening or of >499 mL within 8 weeks of screening<br>• Have any other conditions, which, in the opinion of the Investigator would make the participant unsuitable for inclusion, or could interfere with the participant participating in or completing the study | | | |
| IONIS APO(a)-LRx | NCT02414594 | Phase 1/2a | Inclusion Criteria:<br>• Must have given written informed consent and be able to comply with all study requirements<br>• Healthy males or females aged 18-65 inclusive and weighing ≥ 50 kg at the time of informed consent | | | In the single-ascending-dose cohort, significant dose-dependent reductions in Lp(a) concentration were achieved, with mean reductions at day 30 of 26·2% (SD 5·4) in the 10 mg group, 33·2% (17·5) in the 20 mg |

| | | | | | | |
|---|---|---|---|---|---|---|
| | | | • Females must be non-pregnant and non-lactating, and either surgically sterile or post- menopausal<br>• Males must be surgically sterile, abstinent or using an acceptable contraceptive method<br>• BMI < 35.0 kg/m2<br>• Subjects must have Lp(a) ≥ 75 nanomoles/liter nmol/L (30 mg/dL) at Screening<br>Exclusion Criteria:<br>• Known history of or positive test for human immunodeficiency virus (HIV), hepatitis C or chronic hepatitis B<br>• Treatment with another Study Drug, biological agent, or device within one-month of screening<br>• Regular use of alcohol within 6 months of Screening<br>• Use of concomitant drugs unless authorized by the Sponsor Medical Monitor<br>• Smoking > 10 cigarettes a day | | | singledose group, 43·5% (14·3) in the 40 mg group, 78·6% (21·2) in the 80 mg group, and 85·3% (7·1) in the 120 mg group, versus a 2·8% (21·5) mean increase in the placebo group The eff ects of IONIS-APO(a)-LRx were sustained at day 90, with signifi cant reductions in Lp(a) concentrations of 46% in the 80 mg and 44% in the 120 mg single-dose groups present (p=0·0087 for both). Similarly, dose-dependent reductions in OxPL-apo(a) and OxPL-apoB were achieved in this cohort LDL-C was reduced by more than 20% in the 120 mg group and had a significant treatment difference versus placebo. The 120 mg group also had signifi cant |

| | | | | | | |
|---|---|---|---|---|---|---|
| | | | • Considered unsuitable for inclusion by the Principal Investigator | | | reductions in apoB-100 concentrations versus placebo. In the multiple-ascending-dose cohort, significant dose-dependent reductions in Lp(a) concentration were achieved, with treatment difference versus placebo of 59·4% (95% CI 33·5–79·1) for 10 mg, 72·3% (51·6–87·7) for 20 mg, and 82·4% (67·6–99·8) for 40 mg. The effects of IONIS-APO(a)-LRx were sustained with signifi cant reductions in Lp(a) of 39%, 53%, and 58% in the 10 mg, 20 mg, and 40 mg multidose groups, respectively, at 113 days after the last dose .Significant dose-dependent reductions in OxPL-apo(a) were also present in the multiple-ascending-dose phase, with mean reductions of |

| | | | | | | |
|---|---|---|---|---|---|---|
| | | | | | | up to 72% in the 40 mg group. The eff ects of IONIS-APO(a)-LRx were sustained with signifi cant reductions in OxPL-apo(a) of 34%, 54%, and 66% in the 10 mg, 20 mg, and 40 mg groups, respectively at day 50 (p=0·0127, p=0·0080, and p=0·0007, respectively). For OxPL-apoB, significant reductions were noted at day 36 in the 10 mg, 20 mg, and 40 mg groups In the multiple-ascending-dose cohort, mean LDL-C and apoB-100 concentrations were significantly reduced in the 40 mg treatment group at day 36 |
| AKCEA-APO(a)-LRx, IONIS-APO(a)-LRx, | NCT03070782 | Phase 2 | Key Inclusion Criteria:<br>• Clinical diagnosis of CVD defined as | | | Lp(a)% change: Cohort A participants received 20 |

| | | | | | | |
|---|---|---|---|---|---|---|
| TQJ230 and Pelacarsen | | | documented coronary artery disease, stroke, or peripheral artery disease<br>• Lp(a) plasma level ≥ 60 mg/dL<br>• Must be on standard-of-care preventative therapy for other than elevated Lp(a) CVD risk factors<br>Key Exclusion Criteria:<br>• Within 6 months of Screening: acute coronary syndrome, major cardiac surgery, or stroke/TIA<br>• Within 3 months of Screening: coronary, carotid, or peripheral arterial revascularization, major non-cardiac surgery, or lipoprotein apheresis<br>• Heart failure New York Heart Association (NYHA) class IV | | | milligrams (mg) ISIS 681257, subcutaneous (SC) injection, once every 4 weeks (Q4W), for up to 49 weeks and a maximum of 13 doses. Unit of Measure: percent change :-35(-45 to -22)<br><br>Cohort B participants received 40 mg of ISIS 681257, SC injection, once Q4W, for up to 49 weeks and a maximum of 13 doses. Unit of Measure: percent change :-56 (-63 to -48)<br><br>Cohort C participants received 60 mg of ISIS 681257, SC injection, once Q4W, for up to 49 weeks and a maximum of 13 doses. Unit of Measure: percent |

| | | | | | | |
|---|---|---|---|---|---|---|
| | | | | | | change:-72 (-76 to -67)<br><br>Cohort D participants received 20 mg of ISIS 681257, SC injection, once every 2 weeks (Q2W), for up to 51 weeks and a maximum of 26 doses. Unit of Measure: percent change: -58 (-65 to -50)<br><br>Cohort E participants received 20 mg of ISIS 681257, SC injection, once weekly (QW), for up to 52 weeks and a maximum of 52 doses. Unit of Measure: percent change: -80 (-83 to -76)<br><br>Placebo Participants in each cohort were randomized to receive placebo at a dose-matched volume of study |

| | | | | | | |
|---|---|---|---|---|---|---|
| | | | | | | drug (ISIS 681257). Unit of Measure: percent change : -6 (-21 to 12) |
| Olpasiran (AMG 890) | NCT03626662 | Phase 1 | Inclusion Criteria:<br>• Men and women with ages between 18 and 70 years old, inclusive.<br>• Protocol-defined elevated plasma Lp(a) level.<br>• Body mass index (BMI) greater than or equal to 18 and less than or equal to 40 kg/m2, at screening.<br>• Women must be of non-reproductive potential.<br>• Other Inclusion criteria may apply<br>Exclusion Criteria:<br>• Currently receiving treatment in another investigational device or drug study.<br>• Women who are lactating/breastfeeding or who plan to breastfeed while on study or through 90 days after receiving the last dose of | N/A | N/A | N/A |

| | | | | | | |
|---|---|---|---|---|---|---|
| | | | investigational product (for subjects who withdraw prior to end of study).<br>• History or evidence of a clinically significant disorder, condition or disease that would pose a risk to subject safety or interfere with the study evaluation, procedures or completion.<br>• History or clinical evidence of bleeding diathesis or any coagulation disorder.<br>• History or clinical evidence of peripheral neuropathy.<br>• Other Exclusion criteria may apply | | | |
| Olpasiran (AMG 890) | NCT04270760 | Phase 2 | Inclusion Criteria:<br>• Age 18 to 80 years<br>• Lipoprotein (a) > 150 nmol/L<br>• Evidence of atherosclerotic cardiovascular disease<br>Exclusion Criteria: | N/A | N/A | N/A |

| | | | | | | |
|---|---|---|---|---|---|---|
| | | | • Severe renal dysfunction<br>• History or clinical evidence of hepatic dysfunction<br>• Malignancy within the last 5 years<br>• Currently receiving, or less than 3 months at Day 1 since receiving > 200 mg/day Niacin | | | |
| SLN360 | NCT04606602 | Phase 1 | Inclusion criteria:<br>• Elevated plasma Lp(a) ≥ 150nmol/L.<br>• All subjects must agree to adhere to appropriate contraception requirements.<br>• Subjects must provide written informed consent and be able to comply with all study requirements.<br>• Body mass index of ≥ 18 kg/m2 and ≤ 45 kg/m2.<br>• For the MD part: confirmed history of stable atheroscleroric cardiovascular disease. | Median baseline Lp(a) concentrations were as follows: placebo, 238 (IQR, 203-308) nmol/L; 30-mg SLN360, 171 (IQR, 142-219) nmol/L; 100-mg SLN360, 217 (IQR, 202-274) nmol/L; 300-mg SLN360, 285 (IQR, 195-338) nmol/L; and 600-mg SLN360, 231 | | Maximal median changes in Lp(a) were –20 (IQR, –61 to 3) nmol/L, –89 (IQR, –119 to –61) nmol/L, –185 (IQR, –226 to –163) nmol/L, –268 (IQR, –292 to –189) nmol/L, and –227 (IQR, –270 to –174) nmol/L, with maximal median percentage changes of –10% (IQR, –16% to 1%), –46% (IQR, –64% to –40%), –86% (IQR, –92% to –82%), –96% |

| | | | | | | |
|---|---|---|---|---|---|---|
| | | | Exclusion criteria:<br>• Single Ascending Dose only: any history of clinically overt cardiovascular disease, defined as acute coronary syndromes, myocardial infarction, stable angina, coronary or other revascularization, ischemic stroke or transient ischemic attack and atherosclerotic peripheral arterial disease.<br>• Multiple Dose only: recent history of acute cardiovascular disease events within 6 months of screening (including, but not limited to, acute myocardial infarction, unstable angina, acute stroke and acute limb ischemia).<br>• Moderate or severe hepatic cirrhosis with Child-Pugh grade B or C, or other current or previous liver disease.<br>• Active serious mental illness or psychiatric | (IQR, 179-276) nmol/L. | | (IQR, –98% to –89%), and –98% (IQR, –98% to –97%), for the placebo group and the 30-mg, 100-mg, 300-mg, and 600-mg SLN360 groups, respectively. |

| | | | | | | |
|---|---|---|---|---|---|---|
| | | | disorder, including but not limited to schizophrenia, bipolar disorder, or severe depression requiring current pharmacological intervention.<br>• Any conditions which, in the opinion of the Investigator, would make the subject unsuitable for enrolment in the study or could interfere with the subject's participation in, or completion of the study.<br>• Subjects with previous or current use of medication or therapies significantly affecting Lp(a) level or hormone replacement therapy, unless on a stable dose for ≥ 8 weeks prior to screening<br>• History or clinical evidence of alcohol or illegal drug misuse within the 6 months before screening.<br>• History of multiple drug allergies or history of | | | |

| | | | | | | |
|---|---|---|---|---|---|---|
| | | | allergic reaction to an oligonucleotide or GalNAc, or intolerance to s.c. injections. | | | |
| Mipomersen | NCT00694109 | Phase 3 | Inclusion Criteria:<br><br>Satisfactory completion of dosing in their initial study (Protocol 301012-CS5 [NCT00607373], 301012-CS7 [NCT00706849], 301012-CS17 [NCT00477594], or MIPO3500108 [NCT00794664])<br><br>Exclusion Criteria:<br><br>Had any new condition or worsening of existing condition which in the opinion of the Investigator would make the participant unsuitable for enrollment, or could interfere with the participant participating in or completing the study | | | Percentage LDL levels variation: Mipomersen Sodium 200 mg (for participants weighed ≥ 50 kg) or 160 mg (for participants weighed <50 kg) subcutaneous injection once a week for up to 4 years (depending on participant's consent).<br>Overall Number of Participants Analyzed 141<br>Mean (95% Confidence Interval)<br>Unit of Measure: percent change<br>week 26 (n = 130) -28.5 (-31.9 to -25.1)<br>week 52 (n = 111)-27 (-31.2 to -22.8)<br>week 76 (n = 66)-27.3 (-33 to -21.6) |

| | | | | | | |
|---|---|---|---|---|---|---|
| | | | | | | week 104 (n = 57) -27.9 (-33.9 to -21.8)<br>week 130 (n = 42) -21.9 (-31.1 to -12.7)<br>week 156 (n = 30) -21.4 (-31.2 to -11.7)<br>week 182 (n = 26) -23.6 (-36.6 to -10.6)<br>week 208 (n = 27) -26.3 (-36.4 to -16.2)<br>week 234 (n = 17) -22.5 (-34.3 to -10.6)<br>24 weeks post last dose (n=117) 1.6 (-2.6 to 5.9)<br><br>Percent Change From Baseline in Apolipoprotein B (Apo B)<br><br>Overall Number of Participants Analyzed 141<br>Mean (95% Confidence Interval)<br>Unit of Measure: percent change<br>week 26 (n = 130) |

| | | | | | | |
|---|---|---|---|---|---|---|
| | | | | | | -28.9<br>(-32 to -25.8)<br>week 52 (n = 111)<br><br>-28.1<br>(-32 to -24.2)<br>week 76 (n = 66)<br><br>-30.3<br>(-34.7 to -26)<br>week 104 (n = 57)<br><br>-31.2<br>(-36.5 to -25.9)<br>week 130 (n = 43)<br><br>-29.1<br>(-35.7 to -22.5)<br>week 156 (n = 30)<br><br>-30.2<br>(-38.1 to -22.2)<br>week 182 (n = 26)<br><br>-31.1<br>(-39.9 to -22.2)<br>week 208 (n = 27)<br><br>-33.3<br>(-40.8 to -25.9)<br>week 234 (n = 17)<br><br>-31.4<br>(-38.7 to -24.1)<br>24 weeks post last dose (n=117)<br>-3.46 |

| | | | | | | |
|---|---|---|---|---|---|---|
| | | | | | | (-6.9 to 0)<br>Percent Change From Baseline in Total Cholesterol: Overall Number of Participants Analyzed 141<br>Mean (95% Confidence Interval)<br>Unit of Measure: percent change<br>week 26 (n = 130)<br><br>-21.7<br>(-24.4 to -18.9)<br>week 52 (n = 111)<br><br>-20.4<br>(-23.9 to -16.8)<br>week 76 (n = 66)<br><br>-20.1<br>(-24.6 to -15.5)<br>week 104 (n = 57)<br><br>-19.8<br>(-24.8 to -14.7)<br>week 130 (n = 43)<br><br>-14.9<br>(-22.1 to -7.8)<br>week 156 (n = 30)<br><br>-14.4<br>(-22.3 to -6.6) |

| | | | | | | |
|---|---|---|---|---|---|---|
| | | | | | | week 182 (n = 26)<br><br>-14.3<br>(-25 to -3.5)<br>week 208 (n = 27)<br><br>-16.5<br>(-24.2 to -8.8)<br>week 234 (n = 17)<br><br>-12.5<br>(-21.5 to -3.4)<br>24 weeks post last dose (n=117)<br>1.94<br>(-1.5 to 5.4)<br>Percent Change From Baseline in Non High Density Lipoprotein Cholesterol (Non-HDL-C):<br>Mean (95% Confidence Interval)<br>Unit of Measure: percent change<br>week 26 (n = 130)<br><br>-27.2<br>(-30.4 to -24.1)<br>week 52 (n = 111)<br><br>-25.4<br>(-29.5 to -21.3)<br>week 76 (n = 66) |

| | | | | | | |
|---|---|---|---|---|---|---|
| | | | | | | -25<br>(-30.4 to -19.7)<br>week 104 (n = 57)<br><br>-26.2<br>(-32 to -20.4)<br>week 130 (n = 43)<br><br>-20.7<br>(-29.1 to -12.3)<br>week 156 (n = 30)<br><br>-20<br>(-29.6 to -10.3)<br>week 182 (n = 26)<br><br>-21.7<br>(-34.7 to -8.7)<br>week 208 (n = 27)<br><br>-23.9<br>(-33.7 to -14.1)<br>week 234 (n = 17)<br><br>-19.9<br>(-31.5 to -8.2)<br>24 weeks post last dose (n=117)<br>2.5<br>(-1.8 to 6.7)<br>Percent Change From Baseline in Triglycerides: Overall Number of Participants Analyzed 141 |

| | | | | | | |
|---|---|---|---|---|---|---|
| | | | | | | Mean (95% Confidence Interval)<br>Unit of Measure: percent change<br>week 26 (n = 130)<br><br>-20.1<br>(-33.1 to -1.2)<br>week 52 (n = 111)<br><br>-7.9<br>(-31.5 to 16.9)<br>week 76 (n = 66)<br><br>-10.2<br>(-27.7 to 13.8)<br>week 104 (n = 57)<br><br>-12.5<br>(-37.1 to 7.2)<br>week 130 (n = 43)<br><br>-10.9<br>(-36 to 10)<br>week 156 (n = 30)<br><br>-10.4<br>(-23.8 to 12.7)<br>week 182 (n = 26)<br><br>-12.9<br>(-27.4 to -1.6)<br>week 208 (n = 27)<br><br>-13.9<br>(-40 to 33) |

| | | | | | | |
|---|---|---|---|---|---|---|
| | | | | | | week 234 (n = 17)<br><br>1.3<br>(-15.4 to 15.7)<br>24 weeks post last dose (n=117)<br>2.1<br>(-17.2 to 27.7)<br><br>Percent Change From Baseline in Lipoprotein (a)<br>Overall Number of Participants Analyzed 141<br>Mean (95% Confidence Interval)<br>Unit of Measure: percent change<br>week 26 (n = 130)<br><br>-20.5<br>(-39.3 to -3.6)<br>week 52 (n = 111)<br><br>-19<br>(-33.3 to 0)<br>week 76 (n = 66)<br><br>-17.9<br>(-33.3 to -0.5)<br>week 104 (n = 57)<br><br>-16.6<br>(-36.1 to 0) |

| | | | | | | |
|---|---|---|---|---|---|---|
| | | | | | | week 130 (n = 43)<br><br>-15.8<br>(-31.3 to 0)<br>week 156 (n = 30)<br><br>-9.1<br>(-33.8 to 7.3)<br>week 182 (n = 26)<br><br>-9<br>(-27.2 to 7.6)<br>week 208 (n = 27)<br><br>-9.9<br>(-32.5 to 4.1)<br>week 234 (n = 17)<br><br>-18.3<br>(-31.6 to -4.8)<br>24 weeks post last dose (n=117)<br>0<br>(-6 to 5)<br>Percent Change From Baseline in LDL Particles' Size (Total)<br>Overall Number of Participants Analyzed 140<br>Mean (95% Confidence Interval)<br>Unit of Measure: percent change |

| | | | | | | |
|---|---|---|---|---|---|---|
| | | | | | | week 52 (n = 91)<br><br>-26.77<br>(-32.7 to -20.8)<br>week 104 (n = 47)<br><br>-27.77<br>(-35.3 to -20.3)<br>week 156 (n = 20)<br><br>-25.1<br>(-40.3 to -9.9)<br>week 208 (n = 19)<br><br>-32.65<br>(-44.9 to -20.4)<br>End of treatment (n=139)<br>-22.63<br>(-27 to -18.3)<br>24 weeks post last dose (n=115)<br>6.11<br>(0.8 to 11.5)<br>Percent Change From Baseline in LDL Particles' Size (Large):<br>Overall Number of Participants Analyzed 140<br>Mean (95% Confidence Interval)<br>Unit of Measure: percent change |

| | | | | | | |
|---|---|---|---|---|---|---|
| | | | | | | week 52 (n = 91)<br><br>-5.01<br>(-16.8 to 6.8)<br>week 104 (n = 47)<br><br>-14.32<br>(-27 to -1.6)<br>week 156 (n = 20)<br><br>-27.04<br>(-40.6 to -13.4)<br>week 208 (n = 19)<br><br>-22.67<br>(-41.6 to -3.8)<br>End of treatment (n=139)<br>-2.94<br>(-13.2 to 7.3)<br>24 weeks post last dose (n=115)<br>6.19<br>(-6.1 to 18.5)<br>Percent Change From Baseline in LDL Particles' Size (Medium):<br>Overall Number of Participants Analyzed 140<br>Mean (95% Confidence Interval)<br>Unit of Measure: percent change |

| | | | | | | |
|---|---|---|---|---|---|---|
| | | | | | | week 52 (n = 91)<br><br>-9.50<br>(-31.8 to 12.8)<br>week 104 (n = 47)<br><br>11.09<br>(-42.2 to 64.4)<br>week 156 (n = 20)<br><br>-19.62<br>(-57.3 to 18)<br>week 208 (n = 19)<br><br>-15.82<br>(-62 to 30.4)<br>End of treatment (n=139)<br>-5.65<br>(-30.3 to 19)<br>24 weeks post last dose (n=115)<br>46.92<br>(5.6 to 88.2)<br>Percent Change From Baseline in LDL Particles' Size (Small):<br>Overall Number of Participants Analyzed 140<br>Mean (95% Confidence Interval)<br>Unit of Measure: percent change |

| | | | | | | |
|---|---|---|---|---|---|---|
| | | | | | | week 52 (n = 91)<br><br>-8.79<br>(-33.7 to 16.2)<br>week 104 (n = 47)<br><br>1.72<br>(-43.9 to 47.4)<br>week 156 (n = 20)<br><br>-18.95<br>(-58.8 to 20.9)<br>week 208 (n = 19)<br><br>-27.95<br>(-67.9 to 12)<br>End of treatment (n=139)<br>-5.17<br>(-29.2 to 18.8)<br>24 weeks post last dose (n=115)<br>51.94<br>(7.7 to 96.2)<br>Percent Change From Baseline in LDL Particles' Size (Very Small)<br>Overall Number of Participants Analyzed 140<br>Mean (95% Confidence Interval)<br>Unit of Measure: percent change |

| | | | | | | |
|---|---|---|---|---|---|---|
| | | | | | | week 52 (n = 91)<br><br>-5.05<br>(-32.2 to 22.2)<br>week 104 (n = 47)<br><br>-0.11<br>(-44.4 to 44.2)<br>week 156 (n = 20)<br><br>-18.7<br>(-59.2 to 21.8)<br>week 208 (n = 19)<br><br>-30.77<br>(-69.3 to 7.8)<br>End of treatment (n=139)<br>0.75<br>(-28 to 29.5)<br>24 weeks post last dose (n=115)<br>60.22<br>(7.5 to 112.9)<br>Percent Change From Baseline in HDL Particles' Size (Large):<br>Overall Number of Participants Analyzed 140<br>Mean (95% Confidence Interval)<br>Unit of Measure: percent change |

| | | | | | | |
|---|---|---|---|---|---|---|
| | | | | | | week 52 (n = 89)<br><br>160.8<br>(-38.3 to 359.9)<br>week 104 (n = 47)<br><br>43.23<br>(-12.2 to 98.7)<br>week 156 (n = 20)<br><br>58.26<br>(-38 to 154.6)<br>week 208 (n = 19)<br><br>61.76<br>(-41.6 to 165.2)<br>End of treatment (n=134)<br>121.16<br>(-17.3 to 259.6)<br>24 weeks post last dose (n=110)<br>85.93<br>(-7.3 to 179.1)<br>Percent Change From Baseline in HDL Particles' Size (Medium): Mipomersen<br>Overall Number of Participants Analyzed 140<br>Mean (95% Confidence Interval)<br>Unit of Measure: percent change |

| | | | | | | |
|---|---|---|---|---|---|---|
| | | | | | | week 52 (n = 44)<br><br>154.77<br>(2.8 to 306.8)<br>week 104 (n = 28)<br><br>176.14<br>(-68.6 to 420.9)<br>week 156 (n = 9)<br><br>21.24<br>(-65.1 to 107.6)<br>week 208 (n = 8)<br><br>838.32<br>(-1109.3 to 2785.9)<br>End of treatment (n= 68)<br>388.16<br>(94.5 to 681.8)<br>24 weeks post last dose (n=56)<br>233.78<br>(7.9 to 459.7)<br>Percent Change From Baseline in HDL Particles' Size (Small):<br>Overall Number of Participants Analyzed 140<br>Mean (95% Confidence Interval)<br>Unit of Measure: percent change |

| | | | | | | |
|---|---|---|---|---|---|---|
| | | | | | | week 52 (n = 91)<br><br>1.83<br>(-7.3 to 10.9)<br>week 104 (n = 47)<br><br>-9.81<br>(-17.7 to -2)<br>week 156 (n = 20)<br><br>-14.18<br>(-25.1 to -3.2)<br>week 208 (n = 19)<br><br>-11.47<br>(-20 to -2.9)<br>End of treatment (n= 139)<br>0.44<br>(-6.9 to 7.7)<br>24 weeks post last dose (n=115)<br>8.31<br>(0.6 to 16)<br><br>Percent Change From Baseline in Intermediate Density Lipoprotein Particles' Size<br>Overall Number of Participants Analyzed 140<br>Mean (95% Confidence Interval) |

| | | | | | | |
|---|---|---|---|---|---|---|
| | | | | | | Unit of Measure: percent change<br>week 52 (n = 79)<br><br>-9.9<br>(-45.6 to 25.8)<br>week 104 (n = 40)<br><br>-27.35<br>(-66.5 to 11.8)<br>week 156 (n = 16)<br><br>155.42<br>(-90.1 to 401)<br>week 208 (n = 15)<br><br>32.88<br>(-104 to 169.8)<br>End of treatment (n= 122)<br>24.66<br>(-28.4 to 77.8)<br>24 weeks post last dose (n=101)<br>57.46<br>(15.2 to 99.8)<br><br>Percent Change From Baseline in Very Low Density Lipoprotein (VLDL) Particles' Size (Large) and Chylomicron Particles' Size: |

| | | | | | | |
|---|---|---|---|---|---|---|
| | | | | | | Overall Number of Participants Analyzed 140<br>Mean (95% Confidence Interval)<br>Unit of Measure: percent change<br>week 52 (n = 86)<br><br>109.23<br>(33.7 to 184.8)<br>week 104 (n = 46)<br><br>107.5<br>(-10.2 to 225.2)<br>week 156 (n = 19)<br><br>123.42<br>(-113 to 359.8)<br>week 208 (n = 18)<br><br>241.76<br>(-241.5 to 725.1)<br>End of treatment (n= 132)<br>86.75<br>(28.4 to 145.1)<br>24 weeks post last dose (n=110)<br>90.82<br>(21.8 to 159.9)<br>Percent Change From Baseline in VLDL Particles' Size (Medium): |

| | | | | | | |
|---|---|---|---|---|---|---|
| | | | | | | Overall Number of Participants Analyzed 140<br>Mean (95% Confidence Interval)<br>Unit of Measure: percent change<br>week 52 (n = 88)<br><br>70.81<br>(2.1 to 139.5)<br>week 104 (n = 47)<br><br>97.74<br>(-21 to 216.5)<br>week 156 (n = 20)<br><br>172.46<br>(5.3 to 339.7)<br>week 208 (n = 19)<br><br>98.7<br>(-71.3 to 268.7)<br>End of treatment (n= 136)<br>63.25<br>(12.1 to 114.4)<br>24 weeks post last dose (n=113)<br>99.57<br>(16.8 to 182.3)<br><br>Percent Change From Baseline in VLDL Particles' Size (Small): |

| | | | | | | |
|---|---|---|---|---|---|---|
| | | | | | | Overall Number of Participants Analyzed 140<br>Mean (95% Confidence Interval)<br>Unit of Measure: percent change<br>week 52 (n = 91)<br><br>49.51<br>(-41.1 to 140.4)<br>week 104 (n = 47)<br><br>30.48<br>(-75.1 to 136.1)<br>week 156 (n = 20)<br><br>9.34<br>(-48.3 to 67)<br>week 208 (n = 19)<br><br>-30.36<br>(-49.8 to -10.9)<br>End of treatment (n= 139)<br>31.27<br>(-27.3 to 89.8)<br>24 weeks post last dose (n=115)<br>32.14<br>(-5.3 to 69.6)<br><br>Percent Change From Baseline in Total VLDL Particles' Size and |

| | | | | | | |
|---|---|---|---|---|---|---|
| | | | | | | Chylomicron Particles' Size: Overall Number of Participants Analyzed 140<br>Mean (95% Confidence Interval)<br>Unit of Measure: percent change<br>week 52 (n = 91)<br><br>19.94<br>(-36.7 to 74.6)<br>week 104 (n = 47)<br><br>-14.25<br>(-36.4 to 7.9)<br>week 156 (n = 20)<br><br>3.48<br>(-27.5 to 34.5)<br>week 208 (n = 19)<br><br>-18.66<br>(-43.6 to 6.3)<br>End of treatment (n= 139)<br>12.82<br>(-25.5 to 51.2)<br>24 weeks post last dose (n=115)<br>19.69<br>(3.2 to 36.1)<br><br>Percent Change From Baseline in |

| | | | | | | |
|---|---|---|---|---|---|---|
| | | | | | | Apolipoprotein A-1:<br>Overall Number of Participants Analyzed 141<br>Mean (95% Confidence Interval)<br>Unit of Measure: percent change<br>week 26 (n=130)<br><br>-1.01<br>(-3.8 to 1.7)<br>week 52 (n=111)<br><br>-1.59<br>(-4.7 to 1.6)<br>week 76 (n=66)<br><br>-3.73<br>(-7.9 to 0.5)<br>week 104 (n=57)<br><br>-4.33<br>(-9.1 to 0.4)<br>week 130 (n=43)<br><br>-1.37<br>(-6.1 to 3.4)<br>week 156 (n=30)<br><br>-5.55<br>(-11.2 to 0)<br>week 182 (n=26)<br><br>-3.17 |

| | | | | | | |
|---|---|---|---|---|---|---|
| | | | | | | (-9.4 to 3.1)<br>week 208 (n=27)<br><br>-2.19<br>(-7.2 to 2.8)<br>week 234 (n=17)<br><br>3.68<br>(-3 to 10.3)<br>24 weeks post last dose (n = 117)<br>-0.67<br>(-3.5 to 2.2) |
| ISIS apoC-III Rx | NCT01529424 | Phase 2 | Inclusion Criteria:<br>• Severe hypertriglyceridemia<br>Exclusion Criteria:<br>• HbA1c >/=9.0%, type 1 diabetes, or history of outpatient insulin use for more than 2 weeks in the last year<br>• Body mass index (BMI) >40 kg/m2<br>• History of bariatric surgery or currently on weight loss drugs<br>• Use of oral contraceptives or hormone replacement therapy or statins unless stable for 3 months prior to dosing | | | ISIS 304801 Monotherapy Placebo 100 mg 200 mg 300 mg Parameter (N=16) (N=11) (N=13) (N=11)<br>APOC3, mg/dL :<br>Baseline 22.2 ±7.7 22.4 ±7.7 23.1 ±5.3 22.6 ±6.3<br>Endpoint 21.9 ±10.6 12.0 ±5.2 7.6 ±4.1 4.4 ±2.0<br>% Change 4.2 ±41.7 -40.0 ±32.0* -63.8 ±22.3‡ -79.6 ±9.3<br>LS Mean ±SE 4.2 ±7.5 -39.2 ±9.0‡ -64.2 ±8.3‡ -79.9 ±8.9‡ |

| | | | | | | |
|---|---|---|---|---|---|---|
| | | | • Group 1 and 2 patients: Use of systemic corticosteroids, fibrates, niacin, fish oil or other products containing omega-3 fatty acids within 6 weeks of dosing. Group 3 patients: unable to discontinue use of systemic corticosteroids at least 6 weeks prior to dosing ; use of niacin, fish oil, or other products containing omega-3 fatty acids unless on a stable well controlled dose for at least 30 days prior to screening that is not anticipated to change during the study period. Group 4 patients: unable to discontinue use of systemic corticosteroids at least 6 weeks prior to dosing; use of fibrates niacin, fish oil, or other products containing omega-3 fatty acids unless on a stable well controlled dose for at least 30 days prior to screening that is not | | | Triglycerides, mg/dL:<br>Baseline 522.7 ±369.7 590.6 ±260.3 641.5 ±291.7 559.1 ±224.6<br>Median (IQR) 459 (356, 582) 558 (351, 825) 588 (464, 717) 566 (355, 702)<br>Endpoint 547.1 ±355.4 311.6 ±142.6 234.7 ±163.0 139.5 ±36.4<br>Median (IQR) 456 (343, 640) 277 (226, 394) 180 (157, 262) 120 (115, 185)<br>% Change 20.1 ±72.0 -31.3 ±56.8* -57.7 ±28.3† -70.9 ±14.1‡<br>Median (IQR) 12.1 (-24.6, 28.9) -37.8 (-71.5, -7.0) -70.3 (-79.5, -43.4) -72.2 (-82.8, -67.0)<br>LS Mean ±SE 20.1 ±12.6 -31.2 ±15.1* -58.6 ±13.9‡ -70.9 ±15.0‡<br>Total Cholesterol, mg/dL: |

| | | | | | | |
|---|---|---|---|---|---|---|
| | | | anticipated to change during the study period.<br>• Use of topical corticosteroids, anticoagulants, or drugs or dietary supplements with potential lipid-altering effects unless dose is stable and well controlled for 30 days prior to dosing<br>• Any Screening laboratory values that are out of allowed reference ranges<br>• Inability to comply with protocol or study procedures<br>• Any other significant illness or condition that may adversely affect the subjects participation in the study | | | Baseline 241.1 ±81.2 235.0 ±50.3 240.5 ±62.7 208.9 ±58.7<br>Endpoint 231.8 ±33.3 224.3 ±51.8 223.2 ±34.7 192.4 ±47.3<br>% Change 4.1 ±32.5 -4.0 ±13.7 -1.2 ±15.3 -2.1 ±32.2<br>LS Mean ±SE 4.1 ±6.5 -2.8 ±7.7 -6.1 ±7.1 -2.4 ±7.7<br>HDL-C, mg/dL:<br>Baseline 33.0 ±7.5 31.1 ±4.2 32.3 ±4.1 33.6 ±9.6<br>Endpoint 33.2 ±8.8 38.5 ±8.9 42.9 v8.3 48.3 ±15.1<br>% Change 0.7 ±14.6 26.6 ±38.0 36.2 ±26.0‡ 45.7 ±24.0‡<br>LS Mean ±SE 0.7 ±6.3 28.1 ±7.3† 32.6 ±6.8† 46.0 ±7.3‡<br>Non-HDL-C, mg/dL :<br>Baseline 208.1 ±81.7 203.9 ±53.0 208.2±60.8 175.3 ±60.6 |

| | | | | | | |
|---|---|---|---|---|---|---|
| | | | | | | Endpoint 198.6 ±32.2 185.8 ±49.3 180.3 ±33.6 144.1 ±56.3<br>% Change 6.2 ±40.0 -7.2 ±18.8 -6.8 ±17.0 -11.3 ±38.<br>LS Mean ±SE 6.2 ±7.8 -6.3 ±9.3 -11.8 ±8.6 -11.7 ±9.3<br>LDL-C, mg/dL<br>Baseline 105.1 ±56.3 93.0 ±32.6 80.8 ±25.1 64.6 ±27.7<br>Endpoint 102.1 ±43.4 132.1 ±46.5 134.3 ±35.6 116.5 ±53.6<br>% Change 10.8 ±41.1 48.3 ±39.0* 79.4 ±53.3† 118.3 ±177.3*<br>LS Mean ±SE 10.8 ±23.0 46.9 ±25.8 72.1 ±23.7 110.6 ±25.6†<br>VLDL-C, mg/dL<br>Baseline 110.2 ±80.8 110.9 ±45.9 127.4 ±58.3 110.6 ±54.8<br>Endpoint 96.5 ±43.7 53.7 ±24.6 45.9 ±24.0 27.6 ±7.7 |

| | | | | | | |
|---|---|---|---|---|---|---|
| | | | | | | Change 6.3 ±62.5 -40.0 ±41.8* -56.1 ±27.2† -69.2 ±19.1‡<br>LS Mean ±SE 6.3 ±11.0 -39.5 ±12.7† -57.5 ±11.7‡ -70.0 ±12.7<br>VLDL-APOC3, mg/dL :<br>Baseline 11.6 ±4.9 10.9 ±5.6 13.3 ±5.6 13.9 ±5.8<br>Endpoint 12.1 ±6.2 6.0 ±3.3 3.8 ±3.2 1.5 ±0.9<br>%Change 21.0 ±60.0 -27.3 ±65.6* -66.7 ±26.3‡ -87.6 ±8.6‡<br>LS Mean ±SE 21.0 ±12.1 -29.3 ±13.8† -68.1 ±13.0‡ -85.3 ±13.7<br>Total ApoB, mg/dL :<br>Baseline 117.8 ±38.8 116.3 ±26.1 114.1 ±27.5 100.0 ±20.3<br>Endpoint 115.2 ±23.1 116.7 ±32.4 120.5 ±21.0 101.7 ±41.9<br>Change 3.9 ±26.0 0.5 ±14.9 10.2 ±18.1 1.3 ±36.2 |

| | | | | | | |
|---|---|---|---|---|---|---|
| | | | | | | LS Mean ±SE 3.9 ±6.3 3.6 ±7.4 7.1 ±6.9 1.5 ±7.4<br>ApoB-48, mg/dL:<br>Baseline 0.7 ±0.3 0.9 ±0.6 0.8 ±0.5 0.9 ±0.5<br>Endpoint 1.0 ±0.7 0.6 ±0.3 0.4 ±0.3 0.3 ±0.2<br>% Change 64.5 ±148.6 -20.1 ±45.5* -38.6 ±50.7† -61.1 ±25.5‡<br><br>ISIS 304801 Add-on to Fibrate<br>Placebo 200 mg 300 mg<br>Parameter (N=8) (N=8) (N=10)<br>APOC3, mg/dL :<br>Baseline 19.0 ±5.5 15.5 ±3.6 18.3 ±5.9<br>Endpoint 17.7 ±3.8 6.1 ±2.9 5.1 ±2.1<br>% Change ‡ -2.2 ±25.2 -60.2 ±12.5‡ -70.9 ±13.0‡<br>LS Mean ±SE -2.2 ±6.1 -60.5 ±6.1‡ -71.7 ±5.3‡ |

| | | | | | | |
|---|---|---|---|---|---|---|
| | | | | | | Triglycerides, mg/dL:<br>Baseline 457.0 ±213.5 281.8 ±74.5 393.7 ±227.8<br>Median (IQR) 475 (253, 602) 272 (230, 295) 271 (241, 459)<br>Endpoint 371.7 ±125.0 141.1 ±69.8 134.0 ±60.9<br>Median (IQR)) 338 (290, 394) 120 (103, 172) 116 (91, 191)<br>% Change ‡ -7.7 ±33.8 -51.0 ±13.5† -64.0 ±8.9†<br>Median (IQR) 10.4 (-29.8, 9.7) -43.8 (-63.5, -39.8) -65.4 (-70.7, -54.6)<br>LS Mean ±SE -7.7 ±7.3 -52.3 ±7.4‡ -64.9 ±6.4‡62<br>Total Cholesterol, mg/dL:<br>Baseline 7 228.1 ±61.4 207.6 ±45.9 218.9 ±38.8<br>Endpoint 202.1 ±59.2 188.3 ±28.6 199.6 ±54.7<br>% Change -11.1 ±12.3 -4.0 ±16.1 -7.7 ±23.2 |

| | | | | | | |
|---|---|---|---|---|---|---|
| | | | | | | LS Mean ±SE -11.1 ±6.6 -6.8 ±6.6 -9.0 ±5.7<br>HDL-C, mg/dL:<br>Baseline 6 34.9 ±8.4 36.9 ±9.8 33.9 ±12.3<br>Endpoint 36.3 ±8.6 52.4 ±19.3 51.3 ±19.9<br>% Change 5.9 ±22.2 50.6 ±32.0* 51.80 ±23.7†<br>LS Mean ±SE 5.9 ±9.3 47.5 ±9.5† 49.4 ±8.2†<br>Non-HDL-C, mg/dL :<br>Baseline 193.3 ±65.6 170.8 ±39.8 185.0 ±44.4<br>Endpoint 165.8 ±59.7 135.9 ±23.6 148.3 ±64.2<br>% Change -13.1 ±15.2 -15.2 ±21.0 -18.9 ±28.8<br>LS Mean ±SE-13.1 ±8.1 -18.1 ±8.2 -20.3 ±7.1<br>LDL-C, mg/dL:<br>Baseline.5 ±68.1 118.8 ±37.5 98.0 ±26.2<br>Endpoint 88.2 ±50.9 112.6 ±26.2 120.3 ±62.1 |

| | | | | | | |
|---|---|---|---|---|---|---|
| | | | | | | % Change 5.3 ±35.3 3.54 ±26.4 21.0 ±49.7<br>LS Mean ±SE 5.3 ±14.2 -2.4 ±14.3 18.5 ±12.4<br>VLDL-C, mg/dL :<br>Baseline 98.8 ±49.4 52.0 ±6.8 87.0 ±48.2<br>Endpoint 77.6 ±29.6 23.4 ±11.1 28.0 ±15.3 % Change ‡ -6.5 ±48.8 -54.30 ±22.5* -63.2 ±21.6*<br>LS Mean ±SE‡ -6.5 ±11.4 -54.8 ±11.6† -63.9 ±10.1‡<br>VLDL-APOC3, mg/dL :<br>Baseline 18 9.5 ±3.4 7.5 ±2.2 8.4 ±2.6<br>Endpoint 9.5 ±2.6 2.7 ±1.7 1.9 ±1.0 % %Change 8.4 ±38.0 -66.4 ±15.3‡ -77.2 ±11.4‡<br>LS Mean ±SE 8.4 ±8.2 -66.9 ±8.3‡ -77.9 ±7.2‡<br>Total ApoB, mg/dL : |

| | | | | | | |
|---|---|---|---|---|---|---|
| | | | | | | Baseline 105.3 ±48.3 112.6 ±25.8 106.8 ±27.0<br>Endpoint 95.1 ±44.0 95.8 ±13.4 100.2 ±45.8 % Change -8.4 ±11.9 -9.5 ±22.0 -8.8 ±27.7<br>LS Mean ±SE -8.4 ±7.9 -12.7 ±7.9 -10.4 ±6.9<br>ApoB-48, mg/dL:<br>Baseline 5 0.9 ±0.8 0.5 ±0.3 0.5 ±0.2<br>Endpoint 0.6 ±0.4 0.3 ±0.1 0.3 ±0.2<br>% Change -15.1 ±33.4 -34.4 ±25.7 -33.8 ±20.1<br>Results presented are based on the Per-Protocol population, except for the LS mean percent change from baseline results. Values are the means ± standard deviations in mg/dL, except where indicated. Baseline is defined as the Day -8 value. Endpoint represents the average of the Day 85 and Day 92 |

| | | | | | | |
|---|---|---|---|---|---|---|
| | | | | | | values. Percent (%) change represents from Baseline to Endpoint. IQR denotes interquartile range, Q1 and Q3; LS, least squares; SE, standard error. To convert apolipoprotein values (APOC3, VLDL-APOC3, ApoB, and ApoB-48) to g/L multiply by 0.01; to convert triglyceride (TG) values to mmol/L multiply by 0.0113; to convert cholesterol (C) values to mmol/L, multiply by 0.0259. P-value * |
| Volanesorsen (IONIS-APOCIIIRx) | NCT02658175 | Phase 3 | Inclusion Criteria:<br>• Must give written informed consent to participate in the study (signed and dated) and any authorization required by law.<br>• Able and willing to participate in a 65-week study. | | | Treatment-naïve Group<br>Hide Arm/Group Description:<br>Treatment naïve group included combined group of ISIS 304801-CS7 (CS7-New) study participant and participant on placebo in index |

| | | | | | | |
|---|---|---|---|---|---|---|
| | | | Group 1 and 2:<br>• Satisfactory completion of ISIS 304801-CS6 (NCT02211209) or ISIS 304801-CS16 (NCT02300233) index studies with an acceptable safety profile, per Sponsor and Investigator judgment.<br>Group 3:<br>• Participants who did not participate in the CS6 or CS16 index studies and meet additional inclusion criteria of FCS may enroll in the study.<br>• History of chylomicronemia.<br>• A diagnosis of FCS (Type 1 Hyperlipoproteinemia.)<br>• Fasting triglycerides greater than or equal to (≥)750 milligrams per deciliter [mg/dL] (8.4 millimoles per liter [mmol/L]) at Screening.<br>Exclusion Criteria:<br>• Unwilling to comply with lifestyle | | | studies (ISIS 304801-CS6-Placebo [NCT02211209] and ISIS 304801-CS16-Placebo [NCT02300233]), were to receive 300 mg of volanesorsen as a single SC injection once weekly for Weeks 1-52 of this study. Participants were allowed dose adjustment/dose reduction based on monitoring rules. Following the Week 52 visit, participants had the option of participating in an expanded access program or continuing treatment with 300 mg of volanesorsen as a single SC injection once-weekly for up to an additional 52 weeks (Weeks 53-104) and in France participants, up to an additional 104 weeks for total of |

| | | | | | | |
|---|---|---|---|---|---|---|
| | | | requirements for the duration of the study.<br>Group 1 and 2:<br>• Have any new condition or worsening of existing condition which in the opinion of the Investigator would make the participant unsuitable for enrollment, or could interfere with the participant participating in or completing the study.<br>Group 3:<br>• Diabetes mellitus if newly diagnosed or if hemoglobin A1c (HbA1c)≥ 9.0%.<br>• Active pancreatitis within 4 weeks of screening.<br>• Acute Coronary Syndrome within 6 months of screening.<br>• Major surgery within 3 months of screening.<br>• Treatment with Glybera therapy within 2 years of screening.<br>• Have any other conditions in the | | | 156 weeks of treatment (Weeks 105 to Week 156) of this study until an expanded access program was approved and available in their country.<br>Participants who were not participating in an expanded access program were to enter a 13-week post-treatment evaluation period and in France, participants not continuing treatment were to enter a 26-week post-treatment follow-up period.<br>Overall Number of Participants Analyzed 51<br>Mean (Standard Deviation)<br>Unit of Measure: percent change<br>Percent Change at Month 3 59.8 (37.0)<br>Number Analyzed 47 participants |

| | | | | | | |
|---|---|---|---|---|---|---|
| | | | opinion of the investigator which could interfere with the participant participating in or completing the study. | | | Percent Change at Month 6 -45.5 (42.9)<br>Number Analyzed 49 participants<br><br>Percent Change at Month 12 -36.3 (44.2)<br>Number Analyzed 45 participants<br><br>CS6-Volanesorsen Participants with FCS rolling over from the ISIS 304801-CS6 (NCT02211209) index study after receiving volanesorsen, were to receive 300 mg of volanesorsen as a single SC injection once weekly for Weeks 1-52 of this study. Participants were allowed dose adjustment/dose reduction based on monitoring rules. Following the Week 52 visit, participants had the option of participating in an expanded access |

| | | | | | | |
|---|---|---|---|---|---|---|
| | | | | | | program or continuing treatment with 300 mg of volanesorsen as a single SC injection once-weekly for up to an additional 52 weeks (Weeks 53-104) and in France participants, up to an additional 104 weeks for total of 156 weeks of treatment (Weeks 105 to Week 156) of this study until an expanded access program was approved and available in their country. Participants who were not participating in an expanded access program were to enter a 13-week post-treatment evaluation period and in France, participants not continuing treatment were to enter a 26-week post-treatment follow-up period. |

| | | | | | | |
|---|---|---|---|---|---|---|
| | | | | | | Overall Number of Participants Analyzed 14<br>Mean (Standard Deviation)<br>Unit of Measure: percent change<br>Percent Change at Month 3 -49.2 (34.8)<br>Number Analyzed 14 participants<br>Percent Change at Month 6 -54.8 (23.8)<br>Number Analyzed 13 participants<br><br>Percent Change at Month 12 -35.1 (45.6)<br><br>Number Analyzed 12 participants<br><br><br>CS16-Volanesorsen Participants with FCS rolling over from the ISIS 304801-CS16 (NCT02300233) index study after receiving volanesorsen, were to receive 300 mg |

| | | | | | | |
|---|---|---|---|---|---|---|
| | | | | | | of volanesorsen as a single SC injection once weekly for Weeks 1-52 of this study. Participants were allowed dose adjustment/dose reduction based on monitoring rules. Following the Week 52 visit, participants had the option of participating in an expanded access program or continuing treatment with 300 mg of volanesorsen as a single SC injection once-weekly for up to an additional 52 weeks (Weeks 53-104) and in France participants, up to an additional 104 weeks for total of 156 weeks of treatment (Weeks 105 to Week 156) of this study until an expanded access program was approved and available in their country. |

| | | | | | | |
|---|---|---|---|---|---|---|
| | | | | | | Participants who were not participating in an expanded access program were to enter a 13-week post-treatment evaluation period and in France, participants not continuing treatment were to enter a 26-week post-treatment follow-up period.<br><br>Overall Number of Participants Analyzed 3<br>Mean (Standard Deviation)<br>Unit of Measure: percent change<br>Percent Change at Month 3 -64.9 (9.1)<br>Number Analyzed 3 participants -35.1 (45.6)<br>Percent Change at Month 6 -43.0 (19.7)<br>Number Analyzed 3 participants<br>Percent Change at Month 12 41.6 (36.3) |

| | | | | | | |
|---|---|---|---|---|---|---|
| | | | | | | Number Analyzed 3 participants |
| IONIS APOCIII-LRx | NCT02900027 | Phase I | Inclusion Criteria:<br>• Must have given written informed consent and be able to comply with all study requirements<br>• Healthy males or females aged 18-65 inclusive<br>• Females must be non-pregnant and non-lactating, and either surgically sterile or post- menopausal<br>• Males must be surgically sterile, abstinent or using an acceptable contraceptive method<br>• BMI < 35.0 kg/m2<br>• Subjects must have Fasting TG ≥ 90 mg/dL or ≥ 200 mg/dL depending on Cohort assignment<br>Exclusion Criteria:<br>• Known history of or positive test for human immunodeficiency virus (HIV), hepatitis C or chronic hepatitis B | | | Absolute and mean percent changes in lipids and lipoproteins in the single and multiple ascending dose cohorts<br>AKCEA-APOCIII-LRX, single-dose cohort<br>Pooled placebo (n = 10) 10 mg (n = 6) 30 mg (n = 6) 60 mg (n = 6) 90 mg (n = 6) 120 mg (n = 6)<br>Apo CIII<br>Baseline, mean (SD) 10.4 (2.5) 11.3 (2.3) 8.5 (2.2) 8.8 (3.6) 12.4 (5.3) 14.8 (1.7)<br>Day 15, mean (SD) 13.0 (6.1) 10.7 (1.6) 5.5 (2.2) 3.2 (2.1) |

| | | | | | | |
|---|---|---|---|---|---|---|
| | | | <ul><li>Treatment with another Study Drug, biological agent, or device within one-month of screening</li><li>Regular excessive use of alcohol within 6 months of Screening</li><li>Use of concomitant drugs unless authorized by the Sponsor Medical Monitor</li><li>Smoking > 10 cigarettes a day</li><li>Considered unsuitable for inclusion by the Principal Investigator</li></ul> | | | 2.7 (1.8)<br>1.3 (0.5)<br>Percent change, mean (SD) 23.9 (48.9) −3.6 (14.9)<br>−31.7 (32.5)<br>−64.7 (21.7)<br>−77.9 (12.3)<br>−91.2 (2.5)<br>95% CI for mean<br>−11.1 to 58.8 −19.2 to 12.1 −65.8 to 2.4<br>−87.5 to −41.9 −90.7 to −65.0 −93.8 to −88.6<br>Percent change, median (IQR) 11.0 (4.0 to 23.8) 0.0 (−15.8 to 10.0)<br>−41.7 (−56.5 to −6.7)<br>−72.8 (−80.0 to −47.4)<br>−81.4 (−85.4 to −77.8)<br>−92.4 (−93.1 to −88.6)<br>P-value<br>0.113<br>0.042<br>0.006<br>0.006<br>0.006<br>Triglycerides |

| | | | | | | |
|---|---|---|---|---|---|---|
| | | | | | | Baseline, mean (SD) 134.7 (48.1) 173.3 (67.3) 127.3 (50.1) 139.1 (87.8) 245.4 (130.8) 234.7 (86.6)<br>Day 15, mean (SD) 174.2 (118.0) 147.7 (51.7) 104.2 (33.7) 70.0 (22.9) 68.2 (31.5) 52.0 (11.9)<br>Percent change, mean (SD) 22.2 (44.5) −12.2 (19.5) −10.6 (30.9) −43.0 (19.7) −67.5 (19.0) −76.9 (3.7)<br>95% CI for mean −9.6 to 54.1 −32.6 to 8.2 −43.1 to 21.9 −63.8 to −22.3 −87.5 to −47.6 −80.7 to −73.0<br>Percent change, median (IQR) 9.6 (0.0 to 36.0) −11.6 |

| | | | | | | |
|---|---|---|---|---|---|---|
| | | | | | | (−23.7 to −7.5) −7.3 (−21.4 to 7.9) −42.3 (−57.0 to −29.4) −73.3 (−80.3 to −59.8) −77.1 (−77.8 to −74.6)<br>P-value 0.052 0.137 0.007 0.006 0.006<br>VLDL-C (direct)<br>Baseline, mean (SD) 29.8 (13.3) 35.9 (17.9) 26.6 (5.1) 30.9 (18.5) 59.3 (29.2) 55.4 (15.8)<br>Day 15, mean (SD) 31.4 (21.1) 24.8 (11.8) 25.0 (9.9) 11.0 (6.6) 11.0 (6.7) 17.0 (5.9)<br>Percent change, mean (SD) 5.2 (48.5) −23.4 (31.4) −2.0 (41.9) −65.0 (10.6) |

| | | | | | | |
|---|---|---|---|---|---|---|
| | | | | | | −81.2 (9.1)<br>−68.0 (11.5)<br>95% CI for mean<br>−29.5 to<br>39.9 −56.3 to 9.6<br>−45.9 to<br>41.9 −76.1 to<br>−53.8 −90.8 to<br>−71.6 −80.0 to<br>−55.9<br>Percent change, median (IQR) 0.1 (−41.8 to 41.7)<br>−30.9 (−51.9 to 0.0) −3.0 (−21.4 to 25.5)<br>−64.5 (−68.4 to −56.7)<br>−81.5 (−89.5 to −72.9)<br>−66.8 (−72.0 to −60.0)<br>P-value<br>0.101<br>0.674<br><0.001<br><0.001<br><0.001<br>Non-HDL-C<br>Baseline, mean (SD) 160.7 (24.2)<br>173.2 (36.0)<br>160.8 (31.3)<br>160.4 (30.1) |

| | | | | | | |
|---|---|---|---|---|---|---|
| | | | | | | 163.3 (33.7) 198.4 (15.9)<br>Day 15, mean (SD) 151.0 (39.3) 164.3 (32.5) 148.5 (26.5) 143.3 (39.5) 123.8 (37.1) 147.8 (22.9)<br>Percent change, mean (SD) −6.4 (16.9) −4.5 (10.0) −6.3 (17.2) −11.5 (13.1) −24.4 (15.2) −25.6 (9.2)<br>95% CI for mean −18.5 to 5.7 −15.0 to 5.9 −24.4 to 11.7 −25.3 to 2.3 −40.4 to −8.5 −35.3 to −16.0<br>Percent change, median (IQR) −12.5 (−17.4 to −3.4) −6.3 (−10.1 to 1.3) −7.8 (−22.2 to 0.0) −6.7 (−18.8 to −5.3) −25.7 (−29.3 to −22.1) −24.3 (−33.8 to −23.6) |

| | | | | | | |
|---|---|---|---|---|---|---|
| | | | | | | P-value<br>0.273<br>0.957<br>0.957<br>0.042<br>0.022<br>Total cholesterol<br>Baseline, mean<br>(SD) 213.2 (33.3)<br>218.6 (43.7)<br>205.6 (44.6)<br>198.8 (21.9)<br>203.8 (28.6)<br>238.9 (22.3)<br>Day 15, mean<br>(SD) 204.6 (40.0)<br>213.0 (38.0)<br>201.2 (34.6)<br>194.0 (29.9)<br>188.3 (34.7)<br>211.0 (22.3)<br>Percent change,<br>mean (SD) −3.7<br>(14.8) −2.0 (7.7)<br>−0.5 (15.5)<br>−2.6 (7.7)<br>−7.5 (12.6)<br>−11.5 (8.0)<br>95% CI for mean<br>−14.3 to 6.9<br>−10.0 to 6.0<br>−16.8 to<br>15.7 −10.7 to 5.4<br>−20.7 to 5.8 |

| | | | | | | |
|---|---|---|---|---|---|---|
| | | | | | | −19.8 to −3.1<br>Percent change, median (IQR) −8.1 (−11.9 to −2.2)<br>−3.4 (−5.7 to 4.2) −5.6 (−11.5 to 6.0) −0.5 (−5.2 to 1.1) −8.9 (−13.1 to −5.6)<br>−11.2 (−17.7 to −7.7)<br>P-value<br>0.231<br>0.560<br>0.319<br>0.633<br>0.273<br>LDL-C (ultracentrifugation)<br>Baseline, mean (SD) 131.0 (25.0)<br>137.3 (41.5)<br>134.2 (31.4)<br>129.5 (25.1)<br>104.0 (13.3)<br>143.0 (28.3)<br>Day 15, mean (SD) 119.6 (22.6)<br>139.5 (33.4)<br>123.5 (32.2)<br>132.3 (36.6)<br>112.8 (31.6)<br>130.8 (24.3) |

| | | | | | | |
|---|---|---|---|---|---|---|
| | | | | | | Percent change, mean (SD) −7.7 (14.0) 3.4 (11.3) −7.3 (19.0) 1.8 (18.9) 7.6 (23.1) −7.1 (17.0)<br>95% CI for mean −17.7 to 2.3 −8.5 to 15.2 −27.2 to 12.6 −18.0 to 21.6 −16.6 to 31.9 −25.0 to 10.8<br>Percent change, median (IQR) −9.7 (−12.2 to −6.0) 6.7 (−10.2, 11.3) −4.4 (−22.3 to 8.0) 2.9 (−9.3 to 17.4) −4.1 (−9.7 to 32.2) −9.5 (−17.4 to 0.0)<br>P-value 0.220 0.964 0.291 0.093 0.945<br>ApoB<br>Baseline, mean (SD) 106.9 (22.3) ND ND |

| | | | | | | |
|---|---|---|---|---|---|---|
| | | | | | | ND 99.2 (16.8) 127.4 (12.7)<br>Day 15, mean (SD) 92.0 (19.1)<br>ND ND<br>ND 84.7 (25.2) 94.7 (14.9)<br>Percent change, mean (SD)<br>−13.9 (0.9)<br>−15.9 (13.6)<br>−26.0 (6.8)<br>95% CI for mean<br>−15.4 to −12.4<br>−30.2 to −1.6 −33.1 to −18.9<br>Percent change, median (IQR)<br>−14.1 (−14.5 to −13.3)<br>−13.0 (−26.9 to −4.5)<br>−25.1 (−32.8 to −22.6)<br>P-value<br>1.000<br>0.010 |

| | | | | | | |
|---|---|---|---|---|---|---|
| | | | | | | HDL-C (precipitation)<br><br>Baseline, mean (SD) 52.5 (19.1) 45.4 (12.5) 44.8 (17.8) 38.3 (9.1) 40.5 (8.7) 40.5 (11.9)<br>Day 15, mean (SD) 53.6 (19.2) 48.7 (13.2) 52.7 (20.6) 50.7 (10.8) 64.5 (5.6) 63.2 (11.3)<br>Percent change, mean (SD) 3.7 (14.9) 7.3 (6.4) 18.9 (17.9) 33.5 (13.3) 63.3 (23.2) 61.7 (27.5)<br>95% CI for mean −7.0 to 14.4 0.5 to 14.1 0.2 to 37.7 19.6 to 47.5 38.9 to 87.7 32.8 to 90.6<br>Percent change, median (IQR) 4.0 (−7.8 to 9.1) 7.2 (1.7 to 9.7) 13.9 |

| | | | | | | |
|---|---|---|---|---|---|---|
| | | | | | | (7.3 to 29.7) 32.2 (23.8 to 44.8) 60.6 (43.8 to 79.7) 72.4 (38.3 to 77.5)<br>P-value<br>0.702<br>0.112<br>0.003<br><0.001<br><0.001<br>Lp(a) (nmol/L)<br>Baseline, mean (SD) 17.0 (12.2)<br>ND ND<br>ND 34.8 (36.6) 48.4 (46.0)<br>Day 15, mean (SD) 13.5 (12.0)<br>ND ND<br>ND 33.8 (34.2) 48.8 (52.8)<br>Percent change, mean (SD)<br>−32.6 (33.0)<br>0.7 (26.6) −13.5 (25.9)<br>95% CI for mean<br>−85.2 to 19.9<br>−27.3 to 28.6 −40.8 to 13.7 |

| | | | | | | |
|---|---|---|---|---|---|---|
| | | | | | | Percent change, median (IQR)<br>−18.8<br>(−50.9 to −14.4)<br><br>−8.7<br>(−17.7 to 9.1) −2.8<br>(−36.4 to 0.0)<br>P-value<br><br><br>0.186<br>0.233 |
| Volanesorsen (IONIS-APOCIIIRx) | NCT02300233 | Phase 3 | Inclusion Criteria:<br>1. Body mass index (BMI) ≤ 45 kg/m2<br>2. Fasting Triglycerides (TG) ≥ 500 mg/dL (≥ 5.7 mmol/L) at Screening.<br>3. If on statin or fibrate, participants must be on stable, labeled dose for at least 3 months prior to screening. Participants not receiving these drugs within 4 weeks prior to screening are also eligible.<br><br>Exclusion Criteria:<br>1. Type 1 diabetes mellitus<br>2. Newly diagnosed type 2 diabetes mellitus | | | Percent Change in Fasting Triglycerides (TG) From Baseline to Month 3:<br>Volanesorsen-matching placebo administered subcutaneously once-weekly for 26 weeks.<br>Least Squares Mean (95% Confidence Interval)<br>Unit of Measure: percent change<br>-0.9<br>(-13.9 to 12.2)<br><br>Volanesorsen 300 mg administered subcutaneously once-weekly for 26 |

| | | | | | | |
|---|---|---|---|---|---|---|
| | | | (within 12 weeks of screening) or HbA1c ≥ 9.0% at Screening<br>3. Acute pancreatitis within 3 months of screening<br>4. Acute Coronary Syndrome within 6 months of screening<br>5. Major surgery within 3 months of screening<br>6. Prior exposure to ISIS 304801<br>7. Have any other conditions in the opinion of the investigator which could interfere with the participant participating in or completing the study | | | weeks; or once-weekly for 13 weeks, then bi-weekly for 13 weeks.<br>Least Squares Mean (95% Confidence Interval)<br>Unit of Measure: percent change<br>-71.2<br>(-79.3 to -63.2)<br><br>Percent Change in High-density Lipoprotein-cholesterol (HDL-C) From Baseline:<br><br>Volanesorsen-matching placebo administered subcutaneously once-weekly for 26 weeks.<br>Least Squares Mean (95% Confidence Interval)<br>Unit of Measure: percent change<br>4.4<br>(-5.2 to 14.0)<br><br>Volanesorsen 300 mg administered |

| | | | | | | |
|---|---|---|---|---|---|---|
| | | | | | | subcutaneously once-weekly for 26 weeks; or once-weekly for 13 weeks, then bi-weekly for 13 weeks.<br>Least Squares Mean (95% Confidence Interval)<br>Unit of Measure: percent change<br>61.2<br>(54.2 to 68.3) |
| Volanesorsen (IONIS-APOCIIIRx) | NCT02527343 | Phase 2/3 | Inclusion Criteria:<br>• Must give written informed consent to participate in the study (signed and dated) and any authorizations required by law.<br>• Clinical diagnosis of familial partial lipodystrophy (FPL) plus diagnosis of type 2 diabetes mellitus, hypertriglyceridemia, and fatty liver.<br>• Diagnosis of FPL is based on deficiency of subcutaneous body fat in a partial fashion assessed by physical | | | Participants received volanesorsen-matching placebo as a SC injection once-weekly from Weeks 1 to 52 of the randomized treatment period. Participants were allowed dose adjustment based on monitoring rules.<br>Least Squares Mean (95% Confidence Interval)<br>Unit of Measure: percent change<br>-21.64 |

| | | | | | | |
|---|---|---|---|---|---|---|
| | | | examination and low skinfold thickness in anterior thigh by caliper measurement: men (less than or equal to [≤] 10 millimeters [mm]) and women (≤ 22 mm), and at least 1 of the following:<br>0. Genetic diagnosis of FPL OR<br>1. Family history of FPL or of similar abnormal fat distribution plus 1 Minor Criteria OR<br>2. In the absence of FPL-associated genetic variant or family history, 2 Minor Criteria and body mass index (BMI) less than (<) 35 kilogram per meter square (kg/m^2).<br>• Diabetes not well controlled on antidiabetic therapy with glycated | | | (-60.85 to 17.57)<br><br>Participants received 300 mg of volanesorsen as a SC injection once-weekly from Weeks 1 to 52 of the randomized treatment period. Participants were allowed dose adjustment based on monitoring rules.<br>Least Squares Mean (95% Confidence Interval)<br>Unit of Measure: percent change<br>-88.47<br>(-133.56 to -43.38) |

| | | | | | | |
|---|---|---|---|---|---|---|
| | | | hemoglobin (Hb) HbA1c more than or equal to (≥) 7 percentage (%) to ≤ 12% at Screening.<br>• Hypertriglyceridemia with fasting triglycerides (TG) levels greater than or equal to (≥) 500 milligrams per deciliter (mg/dL) (≥ 5.7 millimoles per liter [mmol/L]) at Screening and Qualification visit, or Fasting TG levels ≥ 200 (≥ 2.26 mmol/L) at both Screening and Qualification Visits for participants who meet the genetic or family history criteria.<br>• Presence of hepatosteatosis (fatty liver), as evidenced by a screening magnetic resonance imaging (MRI) indicating a hepatic fat fraction (HFF) ≥ 6.4%.<br>Exclusion Criteria:<br>• A diagnosis of generalized lipodystrophy.<br>• A diagnosis of acquired partial lipodystrophy. | | | |

| | | | | | | |
|---|---|---|---|---|---|---|
| | | | • Acute pancreatitis within 4 weeks of Screening.<br>• History within 6 months of Screening of acute or unstable cardiac condition.<br>• Low-density lipoprotein cholesterol (LDL-C) more than (>) 130 mg/dL on maximal tolerated statin therapy.<br>• Platelet count < lower limit of normal (LLN).<br>• Treatment with metreleptin within the last 3 months prior to Screening. | | | |
| ARO-APOC3 | NCT03783377 | Phase 1 | Inclusion Criteria:<br>• Women of childbearing potential must have a negative pregnancy test, cannot be breastfeeding and must be willing to use contraception<br>• Willing to provide written informed consent and to comply with study requirements | N/A | N/A | N/A |

| | | | | | | |
|---|---|---|---|---|---|---|
| | | | <ul><li>Normal electrocardiogram (ECG) at screening</li><li>Hypertriglyceridemic patients must have a history of fasting serum triglycerides of at least 300 mg/dL (3.38 mmol/L) at screening or verifiable diagnosis of FCS</li></ul>Exclusion Criteria:<ul><li>Clinically significant health concerns</li><li>Regular use of alcohol within one month prior to Screening</li><li>Use of an investigational agent or device within 30 days prior to dosing or current participation in an investigational study</li><li>Recent use of illicit drugs</li><li>Use of more than two tobacco/nicotine containing or cannabis products per month within 6 months prior to drug administration (applicable only to</li></ul> | | | |

| | | | | | | |
|---|---|---|---|---|---|---|
| | | | Normal Healthy Volunteers)<br>Note: additional inclusion/exclusion criteria may apply, per protocol | | | |
| Inclisiran | NCT03399370 | Phase 3 | Inclusion Criteria:<br>1. Male or female participants ≥18 years of age.<br>2. History of ASCVD (coronary heart disease [CHD], cardiovascular disease [CVD], or peripheral arterial disease [PAD]).<br>3. Serum LDL-C ≥1.8 millimole (mmol)/liter (L) (≥70 mg/dL).<br>4. Fasting triglyceride <4.52 mmol/L (<400 mg/dL) at screening.<br>5. Participants on statins should be receiving a maximally tolerated dose.<br>6. Participants not receiving statins must have documented evidence of intolerance to all doses of at least 2 different statins. | | | Inclisiran sodium 300 milligrams (mg) will be administered as a SC injection on Day 1, Day 90, then every 6 months.<br>Inclisiran Sodium: Inclisiran is a small interfering ribonucleic acid (RNA) that inhibits PCSK9 synthesis.<br>Least Squares Mean (95% Confidence Interval)<br>Unit of Measure: percent change<br>-56.34<br>(-58.35 to -54.34)<br><br>Placebo administered as a subcutaneous injection of sterile saline solution (0.9% sodium chloride in water for injection) on |

| | | | | | | |
|---|---|---|---|---|---|---|
| | | | 7. Subjects on lipid-lower therapies (such as a statin and/or ezetimibe) should be on a stable dose for ≥30 days before screening with no planned medication or dose change during study participation.<br><br>Exclusion Criteria:<br>1. New York Heart Association (NYHA) class IV heart failure.<br>2. Uncontrolled cardiac arrhythmia<br>3. Uncontrolled severe hypertension<br>4. Active liver disease<br>5. Females who are pregnant or nursing, or who are of childbearing potential and unwilling to use at least 2 methods of highly effective contraception (failure rate less than 1% per year) (for example, combined oral contraceptives, barrier methods, approved contraceptive implant, long-term injectable contraception, or | | | Day 1, Day 90, and then every 6 months.<br>Least Squares Mean (95% Confidence Interval)<br>Unit of Measure: percent change<br>1.30<br>(-1.24 to 3.83) |

| | | | | | | |
|---|---|---|---|---|---|---|
| | | | intrauterine device) for the entire duration of the study. Exemptions from this criterion:<br>. Women >2 years postmenopausal (defined as 1 year or longer since last menstrual period) and more than 55 years of age.<br>a. Postmenopausal women (as defined above) and less than 55 years of age with a negative pregnancy test within 24 hours of randomization.<br>b. Women who are surgically sterilized at least 3 months prior to enrollment.<br>6. Males who are unwilling to use an acceptable method of birth control during the entire study | | | |

| | | | | | | |
|---|---|---|---|---|---|---|
| | | | period (such as condom with spermicide).<br>7. Treatment with other investigational products or devices within 30 days or 5 half-lives of the screening visit, whichever is longer.<br>8. Treatment (within 90 days of screening) with monoclonal antibodies directed towards PCSK9<br><br>The above information is not intended to contain all considerations relevant to a participant's potential participation in a clinical trial. | | | |
| Inclisiran | NCT03400800 | Phase 3 | Inclusion Criteria:<br>Participants may be included if they meet all of the following inclusion criteria prior to randomization:<br>1. Male or female participants ≥18 years of age.<br>2. History of ASCVD (coronary heart disease [CHD], cardiovascular disease [CVD], or peripheral arterial disease [PAD]). | | | Inclisiran sodium 300 milligrams (mg) (equivalent to 284 mg inclisiran) in 1.5 millilitres (mL) administered as a subcutaneous injection on Day 1, Day 90, and then every 6 months.<br>Least Squares Mean (95% Confidence Interval)<br>Unit of Measure: Percent change |

| | | | | | | | |
|---|---|---|---|---|---|---|---|
| | | | 3. Serum LDL-C ≥1.8 millimole (mmol)/liter (L) (≥70 mg/dL).<br>4. Fasting triglyceride <4.52 mmol/L (<400 mg/dL) at screening.<br>5. Calculated glomerular filtration rate >30 mL/min by estimated glomerular filtration rate (eGFR) using standardized clinical methodology<br>6. Participants on statins should be receiving a maximally tolerated dose.<br>7. Participants not receiving statins must have documented evidence of intolerance to all doses of at least 2 different statins.<br>8. Subjects on lipid-lower therapies (such as a statin and/or ezetimibe) should be on a stable dose for ≥30 days before screening with no planned medication or dose change during study participation.<br>9. Subjects were willing and able to give | | | -49.3<br>(-51.22 to -47.48)<br><br>Placebo (1.5 mL) administered as a subcutaneous injection of sterile saline solution (0.9% sodium chloride in water for injection) on Day 1, Day 90, and then every 6 months.<br>Least Squares Mean (95% Confidence Interval)<br>Unit of Measure: Percent change<br>4.2<br>(1.62 to 6.69) |

| | | | | | | |
|---|---|---|---|---|---|---|
| | | | informed consent before initiation of any study-related procedures and willing to comply with all required study procedures<br><br>Exclusion Criteria:<br><br>Participants will be excluded from the study if any of the following exclusion criteria apply prior to randomization:<br><br>1. New York Heart Association (NYHA) class IV heart failure.<br>2. Uncontrolled cardiac arrhythmia.<br>3. Uncontrolled severe hypertension.<br>4. Active liver disease.<br>5. Females who are pregnant or nursing, or who are of childbearing potential and unwilling to use at least 2 methods of highly effective contraception (failure rate less than 1% per year) (for example, combined oral contraceptives, barrier methods, approved contraceptive implant, | | | |

| | | | | | | |
|---|---|---|---|---|---|---|
| | | | long-term injectable contraception, or intrauterine device) for the entire duration of the study. Exemptions from this criterion:<br>. Women >2 years postmenopausal (defined as 1 year or longer since last menstrual period) and more than 55 years of age.<br>a. Postmenopausal women (as defined above) and less than 55 years of age with a negative pregnancy test within 24 hours of randomization.<br>b. Women who are surgically sterilized at least 3 months prior to enrollment.<br>6. Males who are unwilling to use an acceptable method of birth control | | | |

| | | | | | | |
|---|---|---|---|---|---|---|
| | | | during the entire study period (such as condom with spermicide).<br>7. Treatment with other investigational products or devices within 30 days or 5 half-lives of the screening visit, whichever is longer.<br>8. Treatment (within 90 days of screening) with monoclonal antibodies directed towards PCSK9.<br><br>The above information is not intended to contain all considerations relevant to a participant's potential participation in a clinical trial. | | | |
| Inclisiran | NCT03705234 | Phase 3 | Inclusion Criteria<br>History or evidence of at least one of the following:<br>• Prior MI; or<br>• Prior ischemic stroke; or<br>• Peripheral artery disease as evident by prior lower extremity artery revascularization or aortic aneurysm repair. | N/A | N/A | N/A |

| | | | | | | |
|---|---|---|---|---|---|---|
| | | | Exclusion Criteria<br>None of the following must be satisfied (based on self-reported medical history):<br>• Acute coronary syndrome or stroke less than 4 weeks before the Screening visit or during the Run-in period;<br>• Coronary revascularization procedure planned within the next 6 months;<br>• Known chronic liver disease;<br>• Current or planned renal dialysis or transplantation;<br>• Previous exposure to inclisiran or participation in a randomized trial of inclisiran;<br>• Previous (within about 3 months), current or planned treatment with a monoclonal antibody targeting PCSK9, or with a drug known to be contra-indicated with inclisiran (none currently known); | | | |

| | | | | | | |
|---|---|---|---|---|---|---|
| | | | • Known to be poorly compliant with clinic visits or prescribed medication;<br>• Medical history that might limit the individual's ability to take trial treatments for the duration of the study (e.g. severe respiratory disease; cancer or evidence of spread within approximately the last 5 years, other than non-melanoma skin cancer; or history of alcohol or substance misuse) or may put the individual at significant risk in the opinion of the investigator (or their authorised deputy) if he/she were to participate in the trial;<br>• Women of child-bearing potential, current pregnancy, or lactation;<br>• Current participation in a clinical trial with an unlicensed drug or device; or<br>• Staff personnel directly involved with the study | | | |

| | | | | | | |
|---|---|---|---|---|---|---|
| | | | and any family member of the investigational study staff. | | | |
| Inclisiran | NCT03159416 | Phase 1 | Inclusion Criteria:<br>• Male and female participants 18 to 80 years of age<br>• Participants should be qualified for inclusion based upon estimated CrCl ranges for normal renal function group and mild, moderate, and severe renal impairment groups<br>Exclusion Criteria:<br>• Participants with acute renal disease and/or history of renal transplant<br>• Urinary incontinence without catheterization<br>• Participants requiring hemodialysis<br>• Participants with LDL-C <60 mg/deciliter (dL) (or less than 1.55 millimoles/liter [mmol/L]) | | | LDL cholesterol level reductions at day 60 were similar for patients with normal renal function (57.6% +/- 10.7%) and participants with mild (35.1%+/- 13.5%), moderate (53.1%+/-21.3%), or severe (49.2%+/- 26.6%) renal impairment |

| | | | | | | |
|---|---|---|---|---|---|---|
| | | | • Participants with Amyloid Kidney (if known by pathology)<br>• Participants with any significant hepatic, cardiac, or pulmonary disease or participants who are clinically nephritic<br>The above information is not intended to contain all considerations relevant to a participant's potential participation in a clinical trial. | | | |
| Inclisiran | Orion 6 | | Inclusion criteria<br><br>All subjects must have satisfied all of the following criteria at the screening visit unless otherwise stated:<br><br>1. Male or female, between 18 and 79 years of age, inclusive<br><br>2. Female subjects were not pregnant (confirmed by a negative pregnancy test within 24 hours of dosing) and nonlactating<br><br>3. The subject, if female, was currently using a double-barrier method of birth control | | | Empty Cell Hepatic function Laboratory parameters* Normal (n = 12) Mild impairment (n = 10) Moderate impairment (n = 6)<br>LDL-C, mg/dL<br><br>Baseline 132.9 (27.4) 97.4 (27.3) 160.0 (177.7)<br>CFB to Day 30 −62.0 (21.4) |

| | | | | | | |
|---|---|---|---|---|---|---|
| | | | or was post-menopausal (defined as at least 1 year without menses; confirmed by follicle-stimulating hormone screen), post-hysterectomy and/or oophorectomy, or surgically sterile (females who had undergone tubal ligation must have used an acceptable method of contraception [ie, barrier method with spermicide])<br><br>4. The subject weighed at least 50 kg (110 pounds) and had a body mass index ≤40 kg/m2 during the screening period<br><br>5. The subject was capable of understanding, and willing to comply with, the protocol and had signed the informed consent document at screening prior to any study-related procedures<br><br>Subjects with normal hepatic function must also have satisfied the following criterion at screening:<br><br>1. Subjects with normal hepatic function were in good health, determined by no clinically significant (CS) | | | −53.9 (32.6)<br>−39.3 (70.6)<br>CFB to Day 30,%<br>−47.1 (13.6)<br>−53.7 (23.5)<br>−22.0 (30.9)<br>CFB to Day 60<br>−68.5 (21.0)<br>−53.2 (27.1)<br>−79.2 (116.4)†<br>CFB to Day 60,%<br>−51.9 (13.1)<br>−53.2 (18.1)<br>−39.7 (16.5)†<br>PCSK9, ng/mL<br><br>Baseline<br>309.7 (52.6)<br>327.9 (94.3)<br>197.1 (132.4)<br>CFB to Day 30<br>−227.4 (60.7) −235.3 (87.4) −95.9 (107.0)<br>CFB to Day 30,%<br>−72.8 (10.7)<br>−71.0 (11.6)<br>−37.8 (27.7)<br>CFB to Day 60<br>−229.7 (55.2) −231.1 |

| | | | | | | |
|---|---|---|---|---|---|---|
| | | | findings from medical history, physical examination, 12-lead electrocardiogram (ECG), vital signs measurements, or clinical laboratory evaluations at screening as assessed by the investigator (or designee)<br><br>Subjects with hepatic impairment must also have satisfied the following criteria at screening:<br><br>1. Subjects had a clinical diagnosis of cirrhosis (stable >3 months) with a documented history of underlying hepatic insufficiency with features of cirrhosis and no acute episodes of illness within 30 days prior to screening and no significant change in disease status from screening to Day –1<br><br>2. Subjects met the criteria for mild or moderate hepatic impairment based on Child-Pugh (CP) classification. Subjects were classified at screening based on CP score and classification was repeated at check-in. If the hepatic function classification for the subject was not the same at the two time points, enrollment of the subject into a hepatic | | | (79.4) −101.7 (122.0)<br>CFB to Day 60,%<br>−73.9 (8.5)<br>−70.3 (11.2)<br>−38.9 (26.0)<br>Total cholesterol, mg/dL<br><br>Baseline<br>207.9 (33.3)<br>173.2 (29.2)<br>231.7 (185.4)<br>CFB to Day 30<br>−56.4 (20.9)<br>−42.8 (38.5)<br>−26.5 (56.4)<br>CFB to Day 30,%<br>−27.1 (8.9)<br>−23.9 (20.1)<br>−6.2 (17.2)<br>CFB to Day 60<br>−67.9 (22.9)<br>−43.1 (42.6)<br>−71.6 (112.4)†<br>CFB to Day 60,%<br>−32.6 (9.3)<br>−24.0 (23.5)<br>−19.7 (15.1)†<br>Triglycerides, mg/dL<br><br>Baseline<br>126.1 (69.2) |

| | | | | | | |
|---|---|---|---|---|---|---|
| | | | category group was based on the CP score at screening<br><br>3. The subject exhibited vital signs within the reference range for their age. Normal was considered a 3-minute sitting blood pressure in the range of 100 mmHg to 160 mmHg systolic, 60 mmHg to 90 mmHg diastolic, and heart rate between 50 and 100 beats per minute. All measurements were performed singly and repeated once if outside the relevant clinical reference range<br><br>Exclusion criteria<br><br>All subjects were excluded from the study if they satisfied any of the following criteria at the screening visit unless otherwise stated:<br><br>1. Known hypersensitivity to inclisiran or related compounds<br><br>2. Previous participation in a study involving inclisiran<br><br>3. Participation in any other investigational study | | | 124.3 (75.5)<br>88.5 (30.5)<br>CFB to Day 30<br>11.1 (43.0)<br>−0.9 (34.5)<br>2.7 (29.0)<br>CFB to Day 30,%<br>9.8 (38.4)<br>7.4 (25.9)<br>0.44 (24.5)<br>CFB to Day 60<br>−5.8 (33.5)<br>−13.3 (78.9)<br>−21.0 (18.8)<br>CFB to Day 60,%<br>−1.4 (26.1)<br>12.6 (104.8)<br>−21.2 (18.9)<br>HDL-C, mg/dL<br><br>Baseline 49.8 (8.6) 51.0 (25.1)<br>53.8 (17.9)<br>CFB to Day 30<br>3.3 (2.9)<br>11.3 (12.4)<br>12.5 (14.8)<br>CFB to Day 30,%<br>7.1 (6.1)<br>23.8 (23.7)<br>21.3 (25.0)<br>CFB to Day 60<br>1.8 (5.5)<br>12.8 (15.1)<br>11.8 (14.1)† |

| | | | | | | |
|---|---|---|---|---|---|---|
| | | | and/or receipt of any investigational drug within 30 days or 5 half-lives prior to dose administration, whichever was longer<br><br>4. The subject had an acute CS illness, other than liver disease and its complications in subjects with mild or moderate hepatic impairment, as determined by the investigator within 30 days prior to the first dose of investigational product<br><br>5. History of uncontrolled or unstable cardiovascular, respiratory, renal, gastrointestinal, endocrine, hematopoietic, psychiatric, and/or neurological disease within 6 months of screening<br><br>6. Subject smoked more than 20 cigarettes (eg, one pack) per day or equivalent (eg, e-vapor cigarette, pipe, cigar, chewing tobacco, nicotine patch, or nicotine gum) and was unable to abstain from the use of tobacco products within 2 hours prior to, and 4 hours after, dose administration | | | CFB to Day 60,%<br>3.4 (9.9)<br>26.8 (27.3)<br>17.4 (25.6)†<br>CFB, change from baseline; HDL-C, high-density lipoprotein cholesterol; LDL-C, low-density lipoprotein cholesterol; PCSK9, proprotein convertase subtilisin/kexin type 9; PD, pharmacodynamic.<br><br>*Data are mean (standard deviation) values.<br>†$n$ = 5. |

| | | | | | | |
|---|---|---|---|---|---|---|
| | | | 7. The subject used vitamins, any alcohol-containing products or medications, green tea, caffeine-containing products or medications, or consumed foods or beverages containing grapefruit or Seville (sour) oranges within 48 hours prior to administration of investigational product<br><br>8. The subject had any clinically important abnormal finding, other than liver disease and its complications in subjects with mild or moderate hepatic function, as determined by the investigator on the medical history, physical examination, 12-lead ECG, or clinical laboratory tests<br><br>9. The subject was unable to understand verbal or written English or any other language for which a certified translation of the informed consent was available<br><br>10. The subject had any other condition that, in the investigator's opinion, prohibited the subject from completing the study or was | | | |

| | | | | | | |
|---|---|---|---|---|---|---|
| | | | not in the best interest of the subject<br><br>11. The subject had a positive human immunodeficiency virus antibody test at screening. Consent and counseling for this procedure was performed according to the site's standard operating procedures<br><br>12. Hemoglobin <9 g/dL and anemia symptoms deemed CS by the investigator or platelets <35,000 at screening or check-in<br><br>13. The addition during the study of medications used to lower LDL-C (eg, statins, ezetimibe, lomitapide, mipomersen, niacin, colesevelam), bile acid absorption inhibitors, or monoclonal antibodies directed towards proprotein convertase subtilisin/kexin type 9 (PCSK9). Note: If a subject began treatment with a monoclonal antibody directed towards PCSK9 after Day 60, they were to be removed from follow-up | | | |

| | | | | | | |
|---|---|---|---|---|---|---|
| | | | 14. Positive urine screen for drugs of abuse (not including cotinine) at screening or check-in, unless result was due to a permitted medication as determined by the medical monitor and/or The Medicines Company (MDCO)<br><br>15. Positive test for alcohol at screening or check-in (as confirmed by alcohol breath or urine test)<br><br>16. Females who were pregnant or nursing, or who were of childbearing potential and unwilling to use at least two methods of acceptable effective contraception (failure rate less than 1% per year [eg, combined barrier methods, approved contraceptive implant, long-term injectable contraception, or intrauterine device]) for the entire duration of the study. Exemptions from this criterion:<br><br>(1) Women >2 years post-menopausal (defined as 1 year or longer since last menstrual period) and more than 55 years of age | | | |

| | | | | | | |
|---|---|---|---|---|---|---|
| | | | (2) Post-menopausal women (as defined above) and less than 55 years of age with a negative pregnancy test within 24 hours of dosing<br><br>(3) Women who were surgically sterilized at least 3 months prior to enrollment<br><br>Subjects with normal hepatic function were excluded from the study if they satisfied any of the following criteria at the screening visit:<br><br>17. The subject had a history of alcohol abuse within the past 12 months, as defined in DSM-IV-TR (Diagnostic and Statistical Manual, 4th Edition) and/or consumed >21 units per week for males and >14 units for females. One unit of alcohol equals 12 oz (360 mL) beer, 1.5 oz (45 mL) liquor, or 5 oz (150 mL) wine<br><br>18. History of uncontrolled or unstable hepatic disease within 6 months of screening<br><br>19. The subject had a positive hepatitis panel, including hepatitis B surface antigen, antihepatitis B core | | | |

| | | | | | | |
|---|---|---|---|---|---|---|
| | | | antibody (HBc; anti-HBc [immunoglobulin G (IgG) + immunoglobulin M, if IgG is positive]), or antihepatitis C virus<br><br>20. The subject had glomerular filtration rate (GFR) <80 mL/min as calculated by using the Cockcroft-Gault equation:<br><br>[1.23 × (140 - age) × (weight in kg)] ÷ (serum creatinine in µmol/L) – if male<br><br>[1.04 × (140 - age) × (weight in kg)] ÷ (serum creatinine in µmol/L) – if female<br><br>21. The subject used any prescription or over-the-counter medication (including oral contraceptives or nutraceuticals such as St. John's wort, ginseng, gingko biloba, kava, or melatonin) within 14 days prior to first dose of investigational product, except for occasional use of acetaminophen (<1 g/day) or other medication approved by MDCO on a case-by-case basis<br><br>Subjects with hepatic impairment were excluded | | | |

| | | | | | | |
|---|---|---|---|---|---|---|
| | | | from the study if they satisfied any of the following criteria at the screening visit:<br><br>22. The subject had a clinical exacerbation of liver disease within the 2-week period before the administration of investigational product (ie, abdominal pain, nausea, vomiting, anorexia, or fever)<br><br>23. The subject had clinically demonstrable massive tense ascites (defined as requiring paracentesis more than once per month)<br><br>24. The subject had evidence of acute viral hepatitis within the past month before entering the study<br><br>25. The subject had a porto-systemic shunt<br><br>26. The subject had evidence of hepatorenal syndrome or GFR <60 mL/min as calculated by using the Cockcroft-Gault equation: | | | |

| | | | | | | |
|---|---|---|---|---|---|---|
| | | | [1.23 × (140 - age) × (weight in kg)] ÷ (serum creatinine in µmol/L) – if male<br><br>[1.04 × (140 - age) × (weight in kg)] ÷ (serum creatinine in µmol/L) – if female<br><br>27. The subject had a history (within the previous 3 months) of alcoholism (consumed >21 units per week for males and >14 units for females. One unit of alcohol equals 12 oz [360 mL] beer, 1.5 oz [45 mL] liquor, or 5 oz [150 mL] wine) and/or drug abuse, and had admitted inability to abstain from alcohol for the period from 48 hours prior to investigational product administration and throughout the study<br><br>Subjects excluded for any of the above reasons were not rescreened for participation at any time if the exclusion characteristic was changed. | | | |

**Supplementary Table S2**

| Target | Aliases for Gene | External Ids for Gene |
|---|---|---|
| Angiopoietin-like protein 3 | • GeneCards Symbol: *ANGPTL3*<br>• Angiopoietin Like 3<br>• ANGPT5<br>• Angiopoietin-Related Protein 3<br>• Angiopoietin 5<br>• ANG-5<br>• Angiopoietin-Like Protein 3<br>• Angiopoietin-Like 3<br>• Angiopoietin-5<br>• FHBL2<br>• ANL3 | • HGNC: 491<br>• NCBI Entrez Gene: 27329<br>• Ensembl: ENSG00000132855<br>• OMIM®: 604774<br>• UniProtKB/Swiss-Prot: Q9Y5C1 |
| Lipoprotein(a). | • GeneCards Symbol: *LPA*<br>• Lipoprotein(A)<br>• Lp(A)<br>• Apolipoprotein(A)<br>• Lipoprotein, Lp(A)<br>• Apo(A) | • HGNC: 6667<br>• NCBI Entrez Gene: 4018<br>• Ensembl: ENSG00000198670 |

| | | |
|---|---|---|
| | • LP<br>• Antiangiogenic AK38 Protein<br>• EC 3.4.21.-<br>• EC 3.4.21.7<br>• EC 3.4.21<br>• AK38<br>• APOA | • OMIM®: 152200<br>• UniProtKB/Swiss-Prot: P08519 |
| Apolipoprotein B | • GeneCards Symbol: *APOB*<br>• Apolipoprotein B<br>• Apolipoprotein B (Including Ag(X) Antigen)<br>• Apolipoprotein B-100<br>• Apolipoprotein B48<br>• Apo B-100<br>• ApoB-100<br>• ApoB-48<br>• LDLCQ4<br>• FCHL2<br>• FLDB | • HGNC: 603<br>• NCBI Entrez Gene: 338<br>• Ensembl: ENSG00000084674<br>• OMIM®: 107730<br>• UniProtKB/Swiss-Prot: P04114 |

| | | |
|---|---|---|
| Apolipoprotein C III | • GeneCards Symbol: *APOC3*<br>• Apolipoprotein C3<br>• Apolipoprotein C-III<br>• Apo-CIII<br>• ApoC-III<br>• APOCIII<br>• Apo-C3<br>• ApoC-3 | • HGNC: 610<br>• NCBI Entrez Gene: 345<br>• Ensembl: ENSG00000110245<br>• OMIM®: 107720<br>• UniProtKB/Swiss-Prot: P02656 |
| Proprotein convertase subtilisin–kexin type 9 (PCSK9) | • GeneCards Symbol: PCSK9<br>• Proprotein Convertase Subtilisin/Kexin Type 9<br>• NARC-1<br>• FH3<br>• Subtilisin/Kexin-Like Protease PC9<br>• HCHOLA3<br>• NARC1<br>• PC9 | • HGNC: 20001<br>• NCBI Entrez Gene: 255738<br>• Ensembl: ENSG00000169174<br>• OMIM®: 607786 |

| | | |
|---|---|---|
| | • Convertase Subtilisin/Kexin Type 9 Preproprotein<br>• Hypercholesterolemia, Autosomal Dominant 3<br>• Neural Apoptosis Regulated Convertase 1<br>• Neural Apoptosis-Regulated Convertase 1<br>• Proprotein Convertase 9<br>• EC 3.4.21.111<br>• EC 3.4.21.-<br>• EC 3.4.21<br>• LDLCQ1<br>• FHCL3 | • UniProtKB/Swiss-Prot: Q8NBP7 |

## Acronyms list

| ACRONYMS | |
|---|---|
| 2VOMe | 2V-O-methyl |
| 2’-MOE | 2’-O-methoxy-ethyl |
| 2’-OMe | 2’-O-methyl |
| AHA | American Heart Association |
| ANGPTL3 | Angiopoietin-like protein 3 |
| ASCVD | Atherosclerotic Cardiovascular Disease |
| ASO | Antisense Oligonucleotides |
| Apo | Apolipoproteins |
| ApoA | Apolipoprotein A |
| ApoB | Apolipoprotein B |
| ApoC-III | Apolipoprotein C III |
| CHD | Coronary Heart Disease |
| CVD | Cardiovascular Disease |
| FCS | Familial Chylomicronemia Syndrome |
| FDA | Food and Drug Administration |
| FH | Familial Hypercholesterolemia |
| GalNAc | N-Acetylgalactosamine |
| HF | Heart Failure |
| HNAs | Hexitol Nucleic Acids |
| LDL - C | Low Density Lipoprotiein Cholesterol |
| LDL | Low Density Lipoprotiein |
| LIPG | Endothelial Lipase |
| LNAs | Locked Nucleic Acids |
| LNPs | Lipid Nanoparticles |
| LPL | Lipoprotein Lipase |
| Lp(a) / LPA | Lipoprotein(a) |
| ncRNA | Non-conding RNA |
| OMO | O-methyl antisense oligonucleotide |
| PCSK9 | Proprotein Convertase Subtilisin–Kexin Type 9 |
| PNA | Peptide Nucleic Acids |
| RISC | RNA-Induced Silencing Complex |
| SAMiRNA | Self-Assembled Micelle Inhibitory RNA |
| siRNA | Double-Stranded Short Interfering RNA |
| TRL | Triacylglycerol (Triglyceride)-Rich Lipoproteins |
| VLDL | Very-Low-Density Lipoprotein |
